# Syntheses Based on 3,4α-Epoxy-1,5,7α,6β(H)-guai-10(14),11(13)-dien-6,12-olide

**DOI:** 10.3390/molecules27061862

**Published:** 2022-03-13

**Authors:** Sergazy Adekenov

**Affiliations:** JSC International Research and Production Holding “Phytochemistry”, Karaganda 100009, Kazakhstan; info@phyto.kz

**Keywords:** sesquiterpene lactones, guaianolide, estafiatin, chemical modification, regio-, stereospecific and chemoselective synthesis, molecular docking, cytotoxicity, antitumor activity, *in vitro*, *in vivo*

## Abstract

The sesquiterpene γ-lactone estafiatin **1**, the molecule of which has a structure of 3,4α-epoxy-1,5,7α,6β(H)-guai-10(14),11(13)-dien-6,12-olide, is characteristic of plants of the genera *Achillea* L. and *Artemisia* L. of the *Asteraceae* family. This article presents the results of chemical modification for three reaction centers of the estafiatin molecule **1**: epoxy cycle, exomethylene group conjugated with γ-lactone carbonyl, and exomethylene group in position C10=C14; and at the same time 33 new derivatives were synthesized, the structures of which were established based on physicochemical constants, spectral data (IR-, PMR-, ^13^C-NMR), and X-ray diffraction analysis. The stereo- and regiospecificity, as well as the chemoselectivity of the reaction based on estafiatin molecule **1**, are discussed. The reactivity of the substrate is significantly influenced by the stereochemistry of its molecule, the nature of the reagent, and the reaction medium. Based on the results of in silico screening, derivatives of estafiatin with high binding energies for both DNA-topoisomerase I and DNA-topoisomerase II were identified. The values of the inhibitory dose of IC_50_ for estafiatin **1** and its derivatives were determined on cell lines of eight types of tumors. *in vivo* experiments of the samples made it possible to establish that estafiatin **1** and its derivatives have pronounced antitumor activity against Pliss lymphosarcoma, Walker’s carcinosarcoma, sarcoma 45, sarcoma-180, alveolar liver cancer PC-1, leukemia P-388 and L-1210, and sarcoma-45 resistant to 5-fluorouracil.

## 1. Introduction

**Figure 1:**
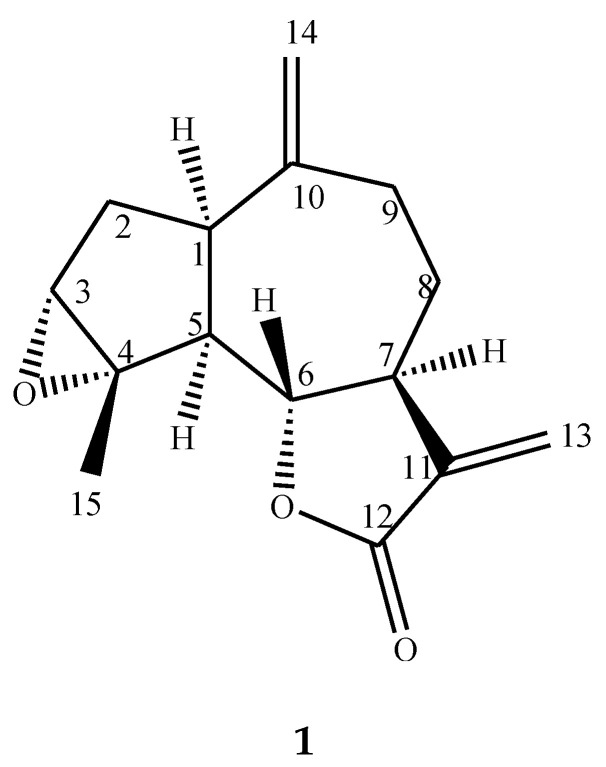
Structure of estafiatin **1**.

Guaianolides constitute one of the largest and most widespread groups of natural sesquiterpene γ-lactones of plant origin. Most of them have pronounced biological activity [1,2,3]. Among the most accessible and promising sesquiterpene lactones of the guaiane type, estafiatin **1** has attracted attention (Figure 1) and was first isolated from *Artemisia mexicana* Willd. ex Spreng., commonly known in Mexico as “Estafiate”, and its extract as an antihelminthic agent [4].

Estafiatin **1** is a major characteristic terpenoid component of *Achillea nobilis* L. and *Stevia alpina* Griseb [5,6,7,8]. *Achillea nobilis* L. is considered renewable plant raw material that has a sustainable operational reserve, which makes it possible to obtain the biologically active sesquiterpene lactone estafiatin on an industrial scale. Within the territory of Central Kazakhstan, on an area of 3916 hectares, the industrial stock of *Achillea nobilis* L. was identified, which amounts to 471.4 tons, with an operational stock of 136.6 tons [9]. 

Estafiatin **1** is an optically active compound that has six asymmetric carbon atoms in its structure. Based on the type of carbon skeleton, estafiatin **1** belongs to bicyclic sesquiterpene γ-lactones of the guaiane type, which have hydroazulene structures contained at C-4-methyl, at C-10-methylene, and at C-7 β-isopropyl groups. The five- and seven-membered carbocycles of estafiatin molecule **1** are articulated in the *cis*-positions, and the articulation of the γ-lactone ring is in the *trans*-position.

Estafiatin **1** has attracted attention due to its biological activity; it exhibits immunomodulatory and anti-inflammatory effects [10], has antiparasitic activity [11,12,13], and is also an inhibitor of the re-initiation of meiosis in amphibian oocytes [14].

Due to its polyfunctionality and chirality, as well as increased reactivity, guaianolide estafiatin **1** is an interesting and promising object for the targeted synthesis of new chiral biologically active compounds. 

According to the literature data [2,4,15], it is known that a number of chemical transformations were carried out with the estafiatin molecule **1**: ozonolysis, catalytic hydrogenation, epoxidation, selective hydrogenation of the C11–C13 double bond, and nucleophilic addition according to the Michael reaction type. It was established that the course of the reactions mainly depends on the structural features of the given guaianolide molecule, i.e., with the presence of an exomethylene group conjugated with the carbonyl of γ-lactone, an exomethylene double bond at position C10=C14, an epoxy group.

In connection with the aforesaid, of undoubted interest is the study of chemical transformations using oxidizing and acidic reagents, as well as the reactions of nucleophilic additions at the reaction centers of the polyfunctional molecule of (−)-estafiatin **1**.

Thus, the guaiane type sesquiterpene γ-lactone estafiatin **1** is considered as a renewable material for chemical modification and production of new potentially pharmacologically active compounds.

## 2. Results

In terms of directed changes in biological activity and the search for new derivatives of estafiatin **1**, we carried out a number of chemical modifications of its molecule, which can be conditionally divided into four groups:

1. reactions on the epoxy cycle of estafiatin;
2. reactions on the trisubstituted double bond;
3. reactions on the exomethylene double bond of the α-methylene-γ-lactone cycle;
4. reactions simultaneously on several functional groups.

### 2.1. Reactions on Epoxy Cycle

One of the directions for the epoxy cycle modification of estafiatin molecule **1** is the synthesis on its basis of a derivative with an α,β-unsaturated keto group, since such a functional group, being a good alkylating center, can purposefully affect the biological activity of the obtained compound.

**Scheme 1:**
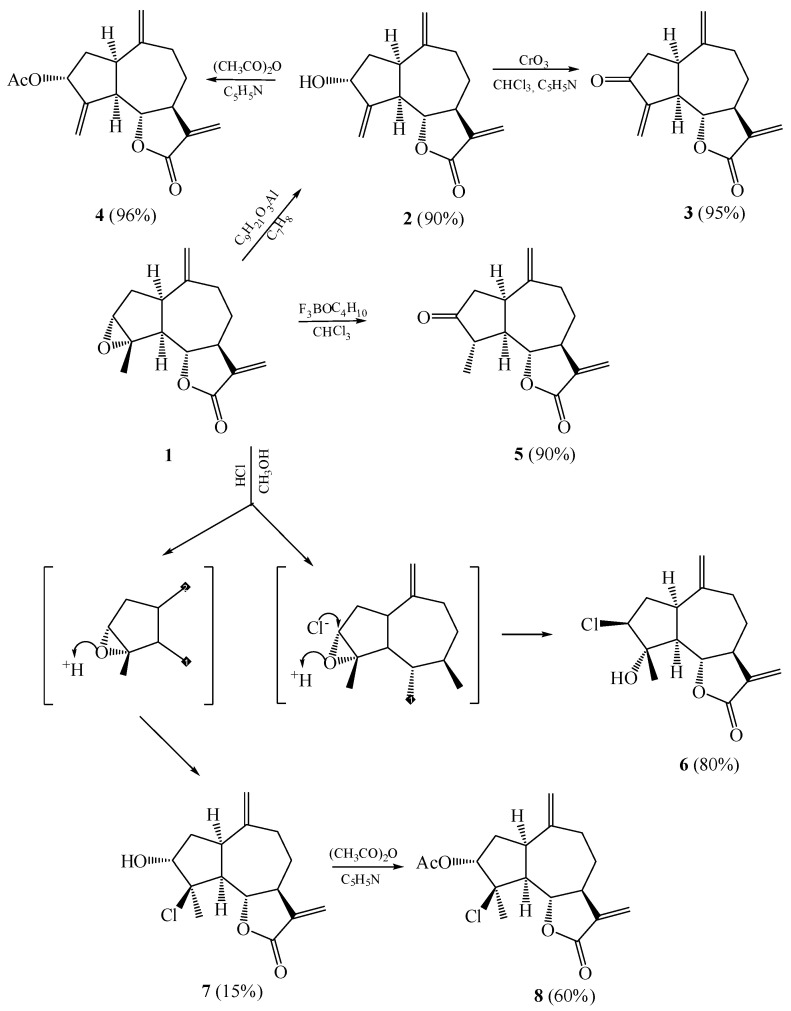
Conversions on the epoxy cycle of estafiatin **1**.

To solve this problem, we carried out isomerization of the α-epoxy ring in molecule **1**, followed by oxidation of the formed secondary hydroxyl group (Scheme 1).

At the first stage, the interaction of estafiatin **1** with aluminum isopropoxide in toluene yielded derivative **2** in the form of a colorless crystalline substance with m.p. 142–144 °C, yield 90%. The data of the IR spectrum of substance **2** characterized the presence of a hydroxyl group in its molecule (3500 cm^−1^). In the PMR spectrum (Table 1) there was a signal of the *gem*-hydroxyl proton-triplet at 4.68 ppm with SSCC of 8 Hz, and two broadened singlets centered at 5.35 and 5.45 ppm, characteristic of the protons of the exomethylene group at C-4.

The above-mentioned data allowed us to propose for molecule **2** the structure of 3α-hydroxy-1,5,7α,6β(H)-guai-4(15),10(14),11(13)-trien-6,12-olide, which turned out to be identical to guaianolide isozaluzanin C, isolated from *Saussurea lappa* [16].

In the next stage (Scheme 1), obtained derivative **2** was oxidized with chromic anhydride in pyridine. This yielded crystalline substance **3** with m.p. 134–135 °C, yield 95%. In its IR spectrum, there was no absorption band for the hydroxyl group, but there was an absorption band for the carbonyl group (1750 cm^−1^). The PMR spectrum (Table 1) showed the signals of the protons of the exomethylene group at C-4-two doublets at 6.23 and 6.28 ppm (1H each, SSCC of 2 Hz), shifted into the weak field by an average of 0.5 ppm due to the influence of the carbonyl group at C-3. The presence of a conjugated α,β-unsaturated keto group in this molecule was also confirmed by the data of the UV spectrum (217 nm, ε 1420), which unambiguously indicated the formation of a molecule 3-keto-1,5,7α,6β(H)-guai-4(15),10(14),11(13)-trien-6,12-olide **3**, identical to 3-keto-4-methylene-*cis*-guaianolide, isolated from *Brachylaena transvaalensis* Hutch. ex Phill. et Schweik [17]. 

Acetylation of isozaluzanin C of **2** with acetic anhydride in pyridine yielded acetyl derivative **4** as a colorless oil, yield 96%. The IR spectrum showed the absorption band of the acetyl group (1745 and 1250 cm*^−^*^1^). The presence of the acetoxy group was also confirmed by the data of the PMR spectrum (Table 1), where there were signals of the protons of the acetyl group-a singlet at 2.02 ppm (3H) and the signal of the gem-acetyl proton as a broadened triplet at 5.62 ppm (SSCC of 6 Hz). 

Based on the data obtained, we came to the conclusion that **4** had the structure of 3α-acetoxy-1,5,7α,6β(H)-guai-4(15),10(14),11(13)-trien-6,12-olide.

In terms of another approach to obtain a 3-keto derivative of estafiatin, we carried out the reaction of molecule **1** with boron trifluoride etherate in chloroform (Scheme 1). At the same time, derivative **5** was obtained, the molecule of which contained a keto group (IR spectrum: 1745 cm*^−^*^1^), which was also confirmed by the data of the PMR spectrum (Table 1), where signals of secondary methyl protons were present at the C-4-a doublet of 1.22 ppm (3H, SSCC of 6 Hz).

Comparison of the physicochemical and spectral data of molecule **5** with the literature data [18,19] made it possible to identify it as guaianolide estafiatone, which has the structure of 3-keto-1,5,7α,4,6β(H)-guai-10(14),11(13)-dien-6,12-olide.

To obtain an analogue of chlorine-containing biologically active guaianolides [20,21,22,23], we studied the interaction of estafiatin **1** with a solution of hydrogen chloride in methanol. This formed a mixture of two substances with R_f_ 0.55 and 0.45. After chromatographic purification of the resulting mixture on silica gel, two regioisomers, **6** and **7**, were isolated with 80% and 15% yields, respectively. In the IR spectrum of **6**, there was an absorption band of the hydroxyl group (3535 cm*^−^*^1^). The PMR spectrum (Table 1) contained a signal at 1.34 ppm in the form of a singlet with an intensity in 3H, characteristic of the gem-hydroxyl methyl group at C-4 and a quartet in the low-field part of the spectrum-4.26 ppm (SSCC of 12.5 and 7 Hz), referring to the protone at C-3, located in the geminal position to the chlorine atom. The PMR spectrum (Table 1) of the second derivative **7** also showed the signals of the protons of the methyl group at C-4-a singlet 1.51 ppm, shifted downfield by 0.17 ppm in comparison with that of the methyl group of molecule **6**, which indicated the influence of the chlorine atom located in the geminal position and the broadened doublet at 3.94 ppm (SSCR of 3 Hz), referring to the gem-hydroxyl proton at C-3. The presence of a hydroxyl group in molecule **7** was also confirmed by the data of the IR spectrum (3536 cm*^−^*^1^). Therefore, we concluded that in the course of this reaction, two isomeric chlorohydrins were formed. 

To confirm the location of the hydroxyl group in the synthesized molecules, acetylation reactions were carried out. The reaction of initial chlorohydrin **6** with acetic anhydride in pyridine at room temperature did not lead to the acetate derivative. This indicated that the hydroxyl group in molecule **6** is tertiary. Acetylation of the second derivative **7** under the same conditions yielded acetate **8** with 60% yield. The presence of the acetyl group was confirmed by the data of the IR spectrum (1756 and 1250 cm*^−^*^1^) and PMR spectrum (Table 1), where the signals of the protons of the acetyl group were observed—a singlet 2.06 (3H) and a doublet centered at 5.33 ppm (SSCC of 4.5 Hz), characteristic of the gem-acetyl proton at C-3.

It should be noted that in the PMR spectrum of acetate **8**, the signals of the protons of the methyl group at C-4 (singlet 1.74 ppm) were shifted downfield by 0.23 ppm in comparison with that of the methyl group of molecule **7**. Such a shift could occur due to the influence of the acetyl group at C-3, which was in the same orientation with the methyl group and thus had an effective descreening effect on the protons of the methyl group at C-4. Therefore, for acetoxy derivative **8**, the structure of 3α-acetoxy- 4β-chloro-1,5,7α,6β(H)-guai-10(14),11(13)-dien-6,12-olide could be suggested. Hence, the structure of 3α-hydroxy-4β-chloro-1,5,7α,6β(H)-guai-10(14),11(13)-dien-6,12-olide was proposed for the initial chlorohydrin **7**; as a consequence, the initial isomer **6** has the structure of 3β-chloro-4α-hydroxy-1,5,7α,6β(H)-guai-10(14),11(13)-dien-6,12-olide.

Based on the structure and stereochemistry of the obtained chlorohydrins **6** and **7**, their formation can be represented as shown in Scheme 1, i.e.*,* the reaction of opening the oxirane ring of the estafiatin molecule **1** was stereoselective, which can be explained by the low conformational mobility of this molecule.

**Figure 2:**
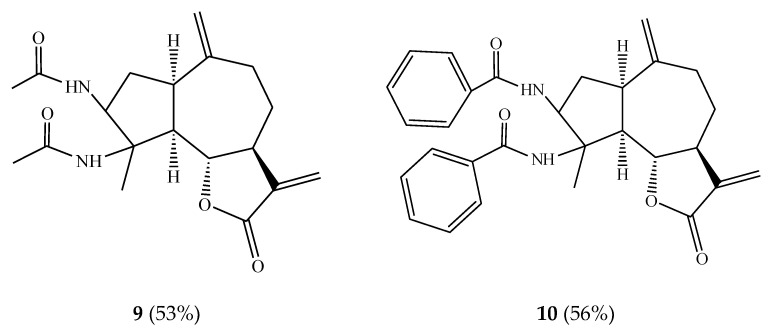
Structures of amino derivatives of estafiatin **9**, **10**.

In order to synthesize practically significant *vic*-diamide derivatives, we carried out the interaction of estafiatin **1** with acetoxy- and benzonitriles in the presence of trace amounts of sulfuric acid at 0 °C. As a result, *vic*-diamides **9** and **10** were obtained with 53% and 56% yields, respectively (Figure 2).

*Vic*-diamide **9** is an optically active crystalline substance of the composition C_19_H_26_O_4_N_2_. The IR spectrum of this molecule contained absorption bands of the C–N bond (1310 cm*^−^*^1^), the carbonyl group of the lactone cycle (1760 cm*^−^*^1^), and the double bond (1635 cm*^−^*^1^). The data of ^1^H NMR-spectra of compound **9** are shown in Table 1.

*Vic*-diamide **10** is also a chiral crystalline substance of the composition C_29_H_30_O_4_N_2_ with a melting point of 108–110 °C (from ethanol) and a specific rotation [α]^20^_D_ +28° (c 0.001; chloroform). The IR spectrum of this molecule contained absorption bands of the C–N bond (1310 cm*^−^*^1^), the carbonyl group of the lactone ring (1770 cm*^−^*^1^), and the double bond (1640 cm*^−^*^1^). The data of the ^1^H NMR spectra of compound **10** are shown in Table 1. As can be seen, the formation of amide groups at the C-3 and C-4 positions apparently occurred due to successive nucleophilic substitution of the epoxy ring by nitriles and hydroxylation followed by in situ tautomerization of intermediate nitriles. The presence of a keto-amine function in the structure of a molecule determines the potential for the development of an antiviral substance on its basis [24].

### 2.2. Reactions on the Exomethylene Group C10=C14

It is known that in the structure of most biologically active natural sesquiterpene γ-lactones, one of the characteristic functional groups is the epoxy group [2]. It is believed that the presence of this group affects the biological activity of the molecules of the compounds of this series. There are various methods of epoxidation, and the choice of the epoxidizing reagent depends mainly on the structure of the substrate, the presence of conjugation of the double bond with the keto or ester group, steric availability of the double bond, and the stability of the molecule in an acidic or alkaline medium [25,26,27].

Based on this, we decided to study the epoxidation reaction on the exomethylene group C10=C14 of estafiatin **1** and its keto derivative **5**.

**Scheme 2:**
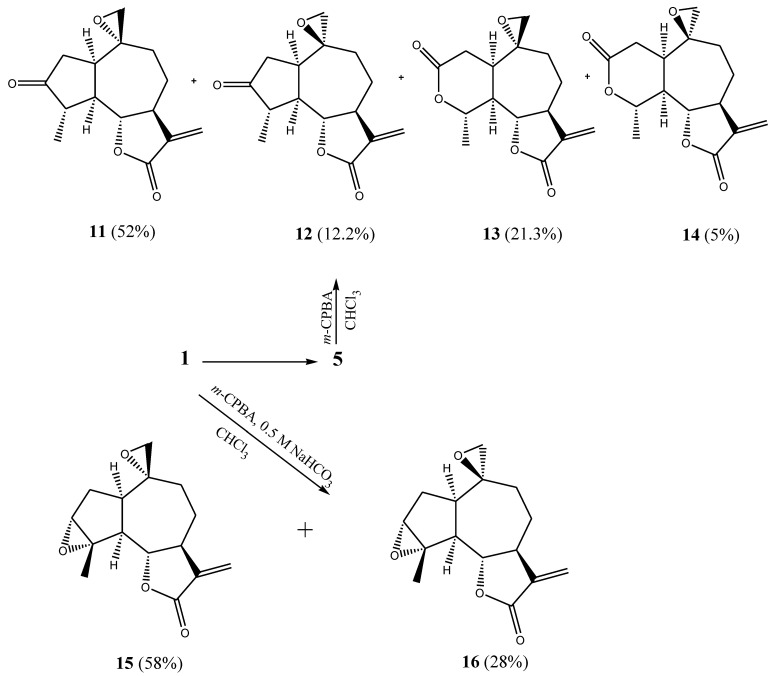
Preparation of epoxy derivatives of estafiatin **11**–**16**.

Epoxidation of estafiatone **5** with m-chloroperbenzoic acid in chloroform formed a mixture of four substances with R_f_ 0.69; 0.55; 0.47; 0.41. Chromatography of the resulting mixture of substances on silica gel isolated derivatives **11**, **12**, **13**, and **14** with yields of 52%, 12.2%, 21.3%, and 5%, respectively (Scheme 2). In the IR spectrum of **11**, there was an absorption band of the epoxy group (1170 cm*^−^*^1^), which was also confirmed by the data of the PMR spectrum (Table 2), where signals of the gem-epoxy C-14 protons were present: two doublets at 2.42 ppm and 2.61 ppm (1H each with SSCC of 4 Hz). The data of the IR spectrum of derivative **12** characterized the presence of an epoxy group (1170 cm*^−^*^1^) in its molecule, as in derivative **11**. In the PMR spectrum of compound **12** (Table 2), the presence of an epoxy ring was confirmed by the presence of signals from gem-epoxy C-14 protons: two doublets, at 2.51 and 2.73 ppm (1H each with SSCC of 4.5 Hz). Moreover, when comparing the PMR-spectra of epoxides **11** and **12**, it was revealed that the signal of the lactone proton of molecule **12** (4.36 ppm) was shifted downfield by 0.22 ppm in comparison with that of epoxide **11**. Such a shift in the signal of the β-oriented lactone proton at C-6 could occur due to the influence of the epoxy group at C10=C14, which was in the same β-orientation, and thus had an effective descreening effect on the lactone proton.

A similar shift in the lactone proton signal was also observed in the presence of a 1,10-epoxy ring in the guaianolide molecule, as in the case of arglabin, arborescine [2].

Based on the above, we came to the conclusion that compound **12** has the structure of 3-keto-10β(14)-epoxy-1,5,7α,4,6β(H)-guai-11(13)-en-6,12-olide, and, therefore, for compound **11** — 3-keto-10α(14)-epoxy-1,5,7α,4,6β(H)-guai-11(13)-en-6,12-olide. In the IR spectrum of the more polar component **13**, absorption bands of the epoxy group (1170 cm^−1^) and carbonyl groups (1780 and 1750 cm^−1^) were observed. The PMR spectrum (Table 2) contained signals of gem-epoxy protons in the form of two doublets, at 2.76 and 2.82 ppm (1H each with SSCC of 4.5 Hz). Moreover, signals of secondary methyl protons were observed at C-4-a doublet 1.50 ppm, shifted downfield by 0.3 ppm in comparison with the secondary methyl signal in the PMR spectrum of the initial estafiatone **5**, as well as the appearance in the low-field part of the spectrum of a signal in the form of a moderate quintet centered at 4.78 ppm (SSCC of 12.5 and 6.5 Hz) assigned to the proton at C-4. It was possible that the downfield shift of the methine proton signal at C-4 was due to the influence of the geminal oxygen atom of the δ-lactone formed during the oxidation of the cyclopentanone part of molecule **5** according to Baeyer-Villiger [28,29]. 

In order to establish the configuration of the epoxy ring, the methyl group at C-4, as well as to elucidate the conformation of the seven-membered ring, an X-ray structural study of the structure of the molecule **13** was carried out. In the structure of molecule **13**, the six-membered ring A is less stressed and more conformationally flexible, the C1-C5 bond length corresponds to 1.536 (8) Å, and the valency angle at C1 and C5 atoms deviate from tetrahedral by no more than 5°. The seven-membered ring B conformation is characterized as a 7,8,9,10-twist-chair (Δ$C_{2}^{8,9}$ = 5.9°). In reality, the replacement of the five-membered carbocycle A by the δ-lactone ring A in **13** did not lead to a noticeable distortion of the conformation of ring B. The conformation of the δ-lactone ring A, *cis*-fused with B (torsion angle H1C1C5H5 −31.3°), strongly distorted 2.4 β-bath (Δ$C_{8}^{1,5}$ = 30.1°). The reason for such a strong distortion of this cycle was the deviation from the unfavorable conformation that was blocked along the C1–C5 bond. The C2C1C5C4 torsion angle was 29.8°, while for an ideal bath conformation, this angle equals 0°. The conformation of the γ-lactone ring, *trans*-fused with cycle B (torsion angle H6C6C7H7 = −163.1°) was a slightly distorted 7α-envelope (Δ$C_{8}^{7}$ = 5.5°). The methyl group at the C-4 atom and the epoxy ring at C10=C14 was in the α-orientation. 

Thus, based on the data obtained for molecule **13**, the structure of 10α(14)-epoxy-1,5,7α,4,6β(H)-guai-11(13)-en-4(3),6(12)-diolide can be proposed.

The data of the IR spectrum of compound **14** characterized the presence of an epoxy cycle (1170 cm^−1^) in its molecule, which was also confirmed by the PMR spectrum (Table 2) by the presence of gem-epoxy proton signals in the form of two doublets, at 2.56 and 2.78 ppm (1H each with SSCC of 4.5 Hz). Moreover, a downfield shift of the lactone proton (4.36 ppm) by 0.19 ppm was observed in comparison with that (4.17 ppm) for molecule **14**, which indicated the effect of the β-oriented epoxy cycle at C10=C14. Along with this, signals of the C-4 methine proton were observed at 4.72 ppm (a quintet with SSCC of 12.5 and 6.5 Hz), which characterized the formation of a δ-lactone ring due to oxidation according to Baeyer-Villiger.

Based on the aforecited data, we came to the conclusion that derivative **14** is an epimer of **13** on the 10(14)-epoxy ring and has the structure of 10β(14)-epoxy-1,5,7α, 4,6β(H)-guai-11(13)-en-4(3),6(12)-diolide.

The interaction of estafiatin **1** with m-chloroperbenzoic acid in chloroform in the presence of a 0.5 M NaHCO_3_ solution yielded two derivatives, **15** and **16**, in the form of colorless crystalline substances with m.p. 158–160 °C and 118–121 °C, respectively (Scheme 2). According to physicochemical constants and spectral data (IR, PMR), the obtained derivatives **15** and **16** turned out to be identical to the known 3α(4),10α(14)-diepoxy-1,5,7α,6β(H)-guai-11(13)-en-6,12-olide and 3α(4),10β(14)-diepoxy-1,5,7α,6β(H)-guai-11(13)-en-6,12-olide, respectively [6]. 

Thus, the presence of α-epoxy cycle and exo-methylene group at C-10 in estafiatin molecule **1** determined the possibilities of studying the stereochemical aspects of the reaction and the synthesis of new biologically active derivatives.

### 2.3. Reactions on Exomethylene Group of γ-Lactone Ring

The α-methylene-γ-lactone fragment, which is responsible for their biological activity, is considered interesting in terms of chemical modification of sesquiterpene lactones. First of all, it allows the biological activity and bioavailability of the initial molecules of sesquiterpene lactones to be increased, which increases interest in their practical application. The prospects of this direction are confirmed by the presence of publications devoted to the search and development of new methods for the chemical modification of sesquiterpene lactones [30,31,32].

Compounds that are analogs of natural phosphates are attracting great attention, since they, as a rule, are chemically more stable than the phosphates themselves and, therefore, may have a prolonged action [33]. In terms of the synthesis of new phosphorus-containing analogs of natural phosphates, potentially possessing high biological activity, dialkylphosphonate derivatives of arteannuin B, grossheimin, were obtained by a method similar to the synthesis of phosphorus derivatives of arglabin [34,35,36].

**Figure 3:**
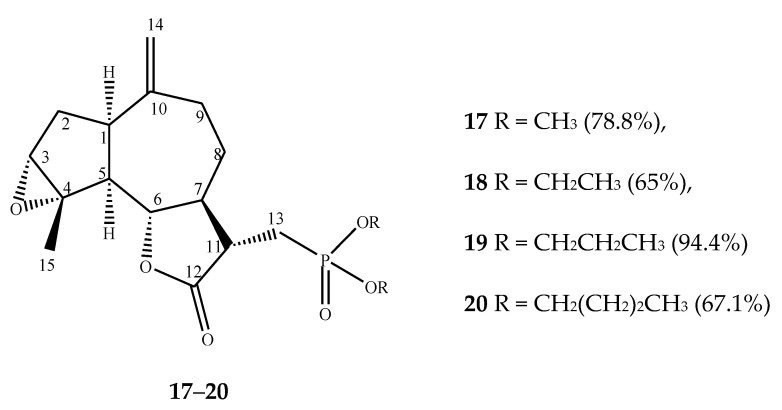
Structures of phosphonate derivatives of estafiatin **17**–**20**.

Based on the sesquiterpene lactone of guaiane type estafiatin **1**, four new dialkylphosphonate derivatives, **17**–**20**, were obtained (Figure 3), the structures of which were unambiguously established using ^1^H and ^13^C NMR spectroscopy, two-dimensional NMR spectroscopy ^1^H-^1^H COSY, ^13^C-^1^H COLOC, and ^31^P-^1^H.

In continuation of the synthesis of estafiatin derivatives, interaction with dimethyl-, diethyl-, dipropyl-, and dibutylphosphites was considered under conditions similar to those described for monoterpene α-enones [37] and sesquiterpene lactone of the cadinane structure arteannuin B [36]. 

The data of the ^1^H NMR and ^13^C NMR spectra of derivatives **17**–**20** are presented in Table 3 and Table 4. The correlation of the PMR and ^13^C NMR signals was performed using 2D spectra of ^1^H–^1^H, ^13^C–^1^H, and ^31^P–^1^H NMR. In the PMR-spectra (Table 3) of derivatives **17**–**20**, the signals of protons of the guaiane skeleton and multiplets of protons H-11 (J_P_^11^_H_ = 21–22), H-13a, and H-13b (J_P_^13^_H_ = 18–20) were complicated by additional cleavage with the phosphorus nucleus by dialkyl phosphite groups. The values of SSCR JPH were in good agreement with the literature data [37]. Because of diastereotopic nature of alkoxy groups, due to the appearance of an additional chiral center at C-11, the signals of the methyl **17** and methylene groups **18**–**20** had different values of the chemical shift, and the splitting of these protons at the phosphorus nucleus led to an additional complication of these protons (the signals of protons at C-1’, C-1”, C-2’, C-2”, C-3’, C-3”, C-4’, and C-4” in Table 3). In all phosphonate derivatives of estafiatin **17**–**20**, the C-11 atom had the R configuration, i.e., protons at C-7 and C-11 were *trans*-oriented (J = 12.0).

The presence of the ^13^C–P bond follows from the data of ^13^C NMR spectra. For example, for derivative **17** in the ^13^C NMR spectrum, the signal of the C-13 nucleus was observed as a doublet with a large (of the order of 145.5 Hz) value of SSCC, which was in good agreement with the values of SSCC J_CP_ from the literature [37]. Due to the influence of the ^31^P nucleus of the dialkylphosphonate group in the ^13^C NMR spectrum, additional cleavage of signals from other carbon nuclei was also observed. So, the signal of the carbon nucleus C=O of the γ-lactone cycle for derivative **17** at 176.4 ppm cleaved in the form of a doublet with SSCC of 13.8 Hz; the signals of the methylene carbon nuclei of the dialkylphosphonate group were also cleaved due to the interaction with the ^31^P nucleus with SSCC of 6.7 Hz for all derivatives **17**–**20**. In addition, an interaction with protons at the C-7, C-11, and C-13 atoms was observed.

Four new dialkylphosphonate derivatives **17**–**20** were obtained for the first time by chemical modification of estafiatin **1**. High chemo- and stereoselectivity of the phosphorylation reaction of the estafiatin molecule **1** was revealed.

Among the most promising directions for the synthesis of the conjugated double bond of the γ-lactone ring is the amination of primary and secondary amines according to the aza-Michael reaction, which makes it possible to obtain water-soluble derivatives, which is practically important for pharmacological and clinical studies of a medicinal substance. The authors of [27,38,39,40,41,42] described the synthesis of a number of amino derivatives of sesquiterpene lactones, such as ludartin, arglabin, grossheimin, alantolactone, parthenolide, helenalin, and ambrosin. Moreover, for amine derivatives of sesquiterpene lactones, pronounced antitumor, anthelmintic, and neuroprotective activity were established [41,43,44,45].

We studied the reactions of nucleophilic additions of various amines (depending on the increase in their basicity) to estafiatin **1**, and it was established that the reactivity of estafiatin **1** differs from other vinyl compounds, primarily in the chemoselectivity of the processes, and depends on the nature of the reacting amines. All reactions were carried out under the same conditions.

**Figure 4:**
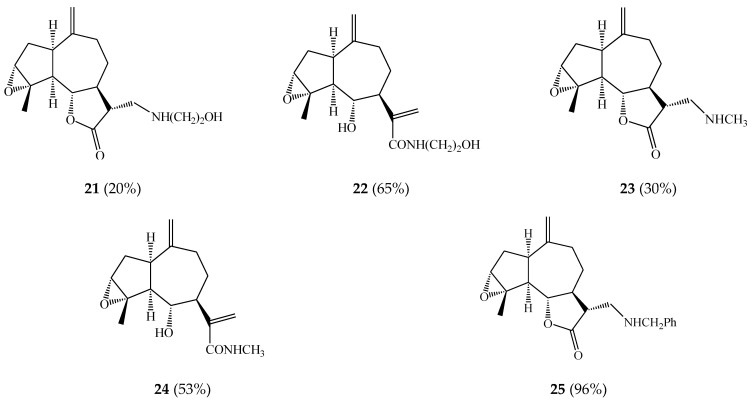
Structures of amino derivatives of estafiatin **21**–**25**.

As such, the reaction of estafiatin **1** with primary aliphatic amines mono-ethanolamine and methylamine, in ethanol at a temperature of 25–30 °C led to the formation of hydroxy-amides **21** and **23** as products of direct nucleophilic addition (aminolysis reaction) with 20% and 30% yields and the products of conjugated addition according to Michael-aminoadducts **22** and **24** with 65% and 53% yields (Figure 4). 

Monoethanolamine derivative **21** is also a crystalline substance of the composition C_17_H_25_O_4_N with a melting point of 135–137° (from ethyl alcohol). The IR spectrum of **21** contained absorption bands of C–N (1130 cm^−1^) and hydroxyl (3530 cm^−1^) groups, as well as absorption bands of the carbonyl group of lactone (1780 cm^−1^). The ^1^H NMR spectrum showed a characteristic signal for protons at C-3 in the form of a broadened singlet at 3.75 ppm, and a signal for the methyl group at C-4 in the form of a singlet at 1.50 ppm was observed. Moreover, the proton H-6 of the lactone was present as a triplet at 3.95 ppm (J = 10 Hz), and the protons of the exomethylene group at H-14 in the form of two broadened singlets at 4.75 and 4.82 ppm; the protons of the H-13 signals were observed as a multiplet at 2.67 ppm, shifted into a strong field. 

Monoethanolamide derivative **22** is a crystalline substance of the composition C_17_H_25_O_4_N with a melting point of 156–158° (from ethyl alcohol). According to the data of the IR spectrum, it was established that molecule **22** contained a hydroxyl group (3530 cm^−1^), and a carbonyl amide group (1660 cm^−1^). In the ^1^H NMR spectrum there was a signal characteristic of protons at C-3 in the form of a broadened singlet of 3.28 ppm, a signal of the C-4 methyl group in the form of a singlet at 1.56 ppm, a signal of heme hydroxyl proton at C-6 as a triplet at 4.0 ppm (J = 10 Hz), a signal of the NH proton in the form of a broadened singlet at 2.14 ppm, and signals of the protons of the exomethylene group in the form of two doublets, at 5.4 and 6.1 ppm (J = 2.5Hz). 

Methylamine derivative **23** has the composition C_16_H_23_O_3_N, is a crystalline substance with m.p. 138–140 °C (ethyl acetate-hexane), and R*_f_* 0.48 (ethyl acetate:benzene, 3:2). The IR spectrum of **23** showed absorption bands of lactone carbonyl (1780 cm^−1^) and C–N (1180 cm^−1^). In the ^1^H NMR spectrum the signal of the H-3 proton was observed, namely a broadened singlet at 2.9 ppm, and the signals of methyl protons at C-4 in the form of a singlet at 1.56 ppm. Due to the polarity of the amino group, the signal of the lactone proton H-6 in the form of a triplet with SSCC of 10 Hz was shifted into a strong field and was observed at 3.18 ppm. Two broadened singlets, at 4.56 and 4.64 ppm, were assigned to exomethylene protons C-14, and the signals of protons at C-13 were observed as a multiplet at 2.50 ppm. In addition, signals of protons of the amino group were present in the form of a broadened singlet at 2.70 ppm and a singlet at 1.90 ppm.

Methylamide derivative **24** is a crystalline substance of the composition C_16_H_23_O_3_N with a melting point of 176–178 °C (from ethyl alcohol). In the IR spectrum of this molecule, absorption bands of the hydroxyl group (3530 cm^−1^) and the amide group (1160, 3400 cm^−1^) were observed. In the ^1^H NMR spectrum a signal of metal protons at C-4 in the form of a singlet at 1.53 ppm, and a signal of the epoxy proton H-3 in the form of a broadened singlet at 2.84 ppm were observed. In addition, two broadened singlets were assigned to the protons of the exomethylene group C-14, at 4.53 and 4.45 ppm; signals from the protons of the exomethylene group C-13 were observed in the form of two doublets, at 5.41 and 6.21 ppm. The signal of the proton H-6-in the form of a triplet was observed at 3.34 ppm. There were signals of the proton of the methylamide fragment in the form of a broadened singlet at 2.52 ppm and a singlet at 1.84 ppm.

The Michael addition was completely stereoselective—only stereoisomers with an α-oriented carbon atom C-13 were formed. Of the two competing reactions, the second, proceeding by the mechanism of conjugated nucleophilic addition, had a slightly higher activation energy, i.e., the carbonyl group was still somewhat more reactive than the C-13 atom. 

By analogy with primary aliphatic amines, one would expect a competing attack by benzylamine mainly at the most reactive carbonyl group of estafiatin **1**; however, we obtained only a conjugated addition product, aminoadduct **25**, with a quantitative 96% yield. The Michael reaction proceeded completely chemoselectively, with the formation of the C-13-α-stereoisomer **25** (Figure 4).

Benzylamine derivative **25** is a crystalline substance of the composition C_22_H_27_O_3_N with m.p. 88–90 °C (ethyl alcohol). In the IR spectrum of this molecule, absorption bands of C–N (1185 cm^−1^), double bond (1640 cm^−1^) were observed. In the ^1^H NMR spectrum of **25**, the signal of the aromatic ring was observed as a broadened singlet at 7.09 ppm. The signals of the protons of the methyl group at C-4 were present as a singlet at 1.53 ppm. A broadened singlet at 2.84 ppm was assigned to the signal of the proton of the epoxy group. The signal of the lactone proton H-6 was observed as a triplet at 3.03 ppm (J = 9Hz). In addition, exomethylene proton signals were observed at 4.50 and 4.56 ppm in the form of a broadened singlet, and the protons of the amino group at C-13 in the form of a singlet at 3.43 ppm. 

Considering that secondary aliphatic amines are, as is known, the most basic and, therefore, more reactive nucleophilic reagents than primary ones, one could more confidently expect their regioselective addition at the carbonyl group of the lactone ring of estafiatin **1**, with the formation of aminolysis products.

**Figure 5:**
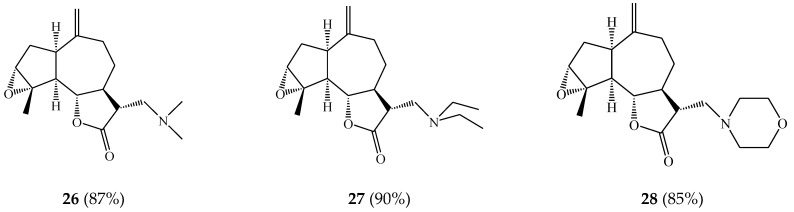

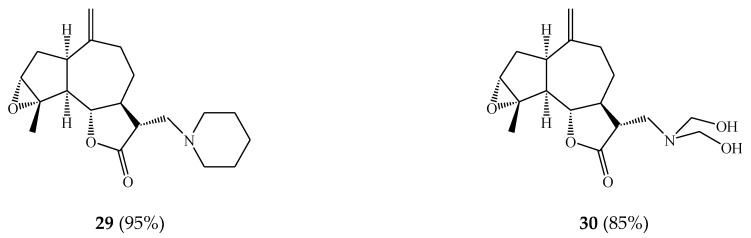
Structures of amino derivatives of estafiatin **26**–**30**.

However, the reactions of estafiatin **1** with dimethylamine, diethylamine, morpholine, piperidine, and diethanolamine in absolute ethyl alcohol medium at 25–30 °C were carried out chemoselectively via the activated C11–C13 double bond (according to the Michael reaction) and led exclusively to quantitative 88–100% yields to aminoadducts **26**–**30** with an α-oriented C-13 atom (Figure 5). 

The obtained amino adducts **26**–**30** were chiral crystalline substances. In all likelihood, the passage of chemoselective nucleophilic addition according to Michael in the reactions of estafiatin **1** with secondary aliphatic and primary fatty aromatic amines is also controlled by the nature of the amines themselves, namely, their hard and soft basic properties. Obviously, the above-mentioned amines belong to boundary bases, such as aniline, pyridine, etc. Therefore, under these conditions, these amines, exhibiting the properties of soft bases, chemoselectively interact with the soft electrophilic carbon atom C-13 of estafiatin **1**, forming only conjugated additions.

**Figure 6:**
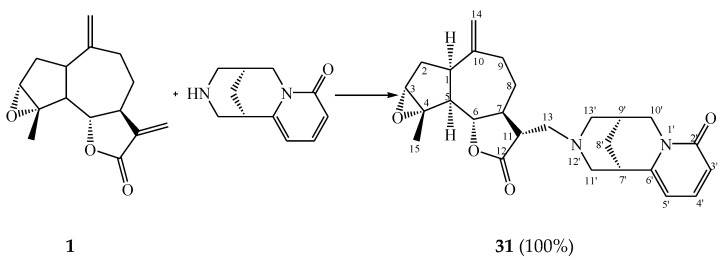
Obtaining of cytisinylestafiatin **31**.

**Figure 7:**
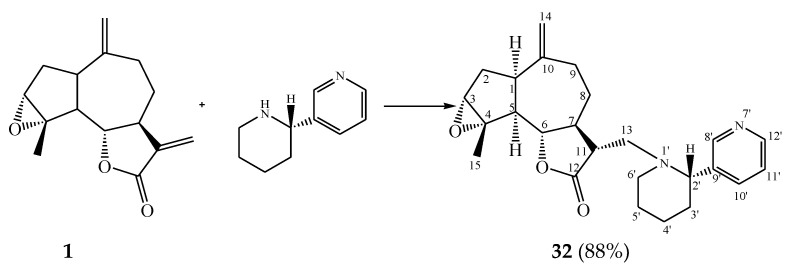
Obtaining of anabasinylestafiatin **32**.

Reaction of estafiatin **1** in a medium of methanol with secondary amines (alkaloids cytisine and anabasine) at room temperature for 24 h led to the formation of cytisinyl and anabasinyl estafiatin derivatives **31**–**32** with 88–100% yields (Figure 6 and Figure 7). 

In the IR spectrum of compound **31**, the following absorption bands were observed: 2973, 2967, 2945, 2934, 2799 (C–H), 1757 (γ-lactone carbonyl), 1652 (C=C), 1579, 1568 (C=C), 1548, 1462, 1450, 1378, 1329, 1300, 1209 (epoxy cycle), 1191 (C–N), 1075, 1048 (CH_2_), 985, 957, 826, 802 (C–O–C).

The signals of the protons of the CH_2_-group of the lactone cycle of estafiatin were characterized as a doublet in the range of δ 4.8–4.9 ppm with SSCC J = 10.24–12 Hz and J = 16–16.4 Hz, and in the ^1^H NMR spectrum of compound **31**, a significant shift of signals into a strong field was observed for protons at C-13, which appeared at 2.47 ppm and 2.72 ppm in the form of a doublet with SSCC of 13.5 and 10.0 Hz and 13.5 and 3.5 Hz, respectively. A single-proton signal in the form of a triplet at 3.81 ppm with J = 10.0 Hz was characteristic of the H-6 lactone proton. In addition, the appearance of a signal at 2.10 ppm could be observed in the form of a doublet doublet with SSCC of 11.0, 8.5 Hz, related to the proton at C-11, which indicated the attachment of the cytisine fragment at C-13.

^13^C NMR spectrum data indicated the presence of twenty-six carbon atoms in the molecule **31**. At the same time, signals were observed that were characteristic of carbon atoms of carbonyl groups in the region of 177.04 and 163.35 ppm, corresponding to the lactone and cytisine carbon atoms, respectively. There was also a shift of the signal of the carbon atom C-13 to the region of a stronger field (58.58 ppm). 

The IR spectrum of molecule **32** contained absorption bands in the region 2984, 2947, 2932, 2912 (C–H), 1774 (C=O γ-lactone), 1633 (C=N), 1591, 1577, 1568 (C=C), 1453, 1443, 1427, 1327, 1303, 1209 (epoxy cycle), 1199 (C–N), 1054, 1024, 1003 (CH_2_), 985, 961, 826, 802 (C–O–C).

The absence in the PMR spectrum of the signal of the protons of the exomethylene group C11–C13 for compound **32** confirmed the addition of the anabasine alkaloid to the exomethylene double bond of γ-lactone. The proton signals of compound **32** at the C-13 atom appeared in the region of 2.31 ppm and 2.66 ppm in the form of doublet doublets with SSCC of 13.53 Hz, 2.72 Hz and 13.53 Hz, and 6.87 Hz, respectively. Single-proton signal H-6 in the form of a triplet at 3.95 ppm with J = 10.5 Hz was characteristic of the lactone proton. ^13^C NMR spectrum data indicated the presence of twenty-five carbon atoms in the molecule **32**. In this case, a signal characteristic of the γ-lactone carbonyl in the region of 178.05 ppm was observed, as well as olefin signals of the anabasine fragment at 123.65 ppm, 135.56 ppm, 140.30 ppm, 148.76 ppm, and 149.61 ppm. There was also a shift of the signal of the carbon atom C-13 to the region of a stronger field. 

The reactions carried out on the exomethylene group of the γ-lactone cycle of estafiatin **1** with primary and secondary amines proceeded chemoselectively with quantitative yields of the corresponding amino derivatives.

Thus, synthesized new potentially biologically active estafiatin **1** derivatives are of interest for studying their pharmacological activity.

### 2.4. Synthesis of Dihalocyclopropane Derivatives of Estafiatin

Dihalocarbene derivatives of sesquiterpene lactones were first described by Salazar and Diaz [46], who synthesized a number of difluorocarbene derivatives of pseudoguaianolides using sodium difluoroacetate as a source of difluorocarbene.

The reaction of cycloaddition of various olefins to dihalocarbens formed under the conditions of phase transfer catalysis is a convenient method for the preparation of dihalocyclopropanes. The use of the dichlorocyclopropanation reaction made it possible to carry out a complex transformation of isoalantholactone with the formation of four chlorine-containing compounds, the yield of which depended on the duration of the reaction [47].

Earlier it was established that dibromocarbene, in contrast to dichlorocarbene, gives an easily isolated product of addition to the exomethylene group of the arglabin molecule [48]. In order to establish the features of the addition of dihalogenocarbens to natural butenolide molecules, we carried out similar reactions with another guaianolide, estafiatin **1**. 

**Figure 8:**
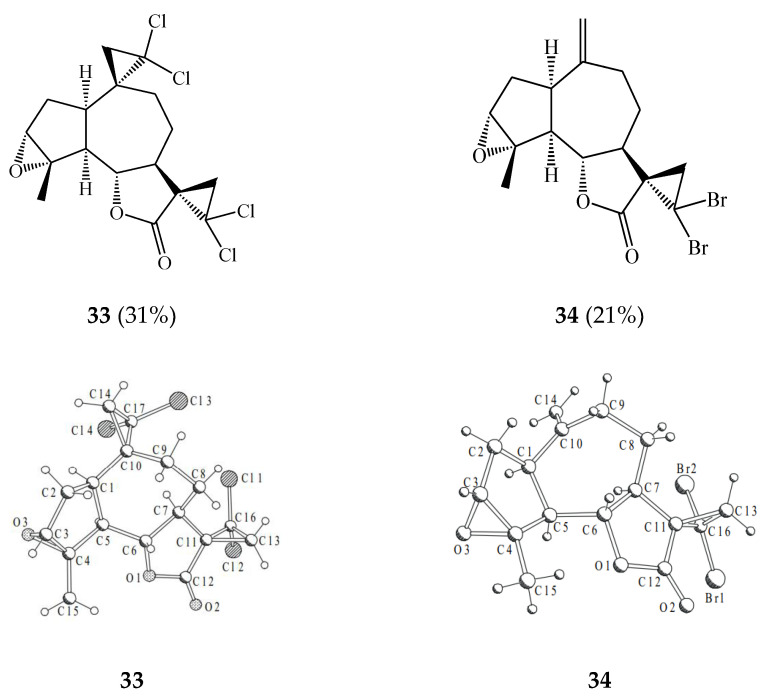
Spatial structure of molecules **33** and **34** according to X-ray diffraction analysis data.

During the interaction of lactone **1** with dichlorocarbene under the conditions of interphase catalysis using dicyclohexyl-18-crown-6, we managed to isolate one substance (yield 31%), which has the composition C_17_H_18_O_3_Cl_4_ (high resolution mass spectrometry) and corresponds to the addition of two dichlorocarbene molecules to the initial molecule **1**. The structure of molecule **33** was established by X-ray diffraction analysis (Figure 8).

When lactone **1** interacted with bromoform under the conditions of d-bromocarbene generation [49], a mixture of products was formed, from which the **1** dibromocyclopropane derivative of estafiatin was isolated with a yield of 21%; the structure of the molecule was expressed by structural formula **34**, also established by X-ray diffraction analysis (Figure 8).

The bond lengths of the studied molecules **33** and **34** were usual [50] within the limits of errors. The lactone ring in molecules **33** and **34** had an envelope conformation with the C6 atom yielding by 0.327 (3) and 0.29 (1) Å from the plane of the rest of the cycle atoms. The conformation of the seven-membered ring was also the same for molecules **33** and **34**, which, according to the Kremer–Pople parameters, can be characterized as intermediate between a chair and a twist-chair. According to the Cambridge Structural Database [51], practically the same seven-membered ring conformation was found, for example, in the bahia I lactone [52] and in β-epoxyestafiatin [6]. The conformation of the five-membered ring in **33** was close to the shape of an envelope with an atom yielding by 0.432 (4) Å from the plane of the remaining atoms, and in **34** it was closer to the twist form with a yielding of C1 and C2 atoms by −0.24 (2) and 0.16 (2) Å, respectively.

In the crystal of **33,** molecules were linked by weak C14-H...O3 interactions (distance H...O 2.40 Å, angle C-H...O 152°) into zigzag chains along the *a* axis. The interactions of C6-H...O2 (H...O 2.54 Å, C-H...O 116°) and C=O2...C12 [O...C 2.921(4) Å] united the chains into layers parallel to the *ab* plane. Of noted are the interlayer interactions of C15-H...Cl1 with the distance H...Cl 2.81 Å and angle C-H...O 163°. In crystal **34**, layers of molecules were not visible; of note are the following abbreviated [53] intermolecular contacts: C3-H...C12, with a distance H...C 2.76 Å, Br2...O3 3.25 (1) Å. 

Thus, upon the interaction of lactone **1** with dichlorocarbene, as well as with bromoform under the conditions of dibromocarbene generation, 3α(4)-epoxy-10(14),11(13)-bis-(dichloro-cyclopropano)-1,5,7α,6β(H)-guai-6,12-olide **33** and 3α(4)-epoxy-11(13)-dibromo-cyclopropano-1,5,7α,6β(H)-guai-10(14)-en-6,12-olide **34** were isolated.

## 3. Biological Activity

Previously [10], experimental results showed that estafiatin **1** was able to modulate activation-induced Ca2^+^ mobilization in Jurkat T-cells and inhibit anti-CD3-induced intercellular Ca2^+^ ([Ca2^+^]i) mobilization in Jurkat cells. In addition to inhibiting extracellular signal regulated kinase (ERK) 1/2 phosphorylation in activated Jurkat cells with IC_50_ = 15.4 mM, molecule **1** inhibited phosphorylation of p53, AMPKa1, CREB, and p27 induced by TCR activation in Jurkat cells. 

When studying the antiparasitic activity of estafiatin **1**, it was determined that molecule **1** exhibited activity and selectivity against *Leishmania braziliensis* promastigotes at a concentration of IC_50_ = 1.0 μg/mL [11]. In addition, compound **1** demonstrated *in vitro* activity against infectious and intracellular forms of *Trypanosoma cruzi* [12]. 

The exomethylene group of γ-lactone and the epoxide function in the structure of estafiatin **1**, interacting with the SH group of the enzyme, act on the Myt1 kinase, thereby inhibiting the re-initiation of meiosis in amphibian *Rhinella arenarum* oocytes [14].

Our study of the antitumor activity of estafiatin **1** and its derivatives showed that the main pharmacophore centers in molecule **1** were the exomethylene group conjugated with the carbonyl group of γ-lactone, the oxirane ring at C3–C4, and the methylene function at C10=C14, interacting with active centers of enzymes, such as farnesyl protein transferase, topoisomerases-I and -II.

When studying the antitumor activity of samples of estafiatin 1 and derivatives synthesized on its basis, molecular docking for DNA-topoisomerase I and II receptors was carried out at the beginning (Table 5).

As a result of the molecular docking, it was revealed that the best values of the binding energy with DNA topoisomerase I were shown by compounds **2** (−7.168 kcal/mol), **4** (−7.041 kcal/mol), and **6** (−7.013 kcal/mol), and with DNA topoisomerase II, compounds **2** (−8.542 kcal/mol), **3** (−8.013 kcal/mol), **5** (−8.286 kcal/mol), and **13** (−8.458 kcal/mol).

In a complex with DNA topoisomerase I, relatively good indicators of ligand efficiency (LE ≥ 0.3) had **1** and **5** (LE is 0.36), **2** (LE is 0.40), **3** and **6** (LE is 0.37), and in a complex with DNA topoisomerase II-compounds **1** (LE is 0.43), **2** (LE is 0.47), **3** (LE is 0.45), and **5** (LE is 0.46). 

Compound **2** of all studied samples showed the best values of the estimated binding energy with DNA topoisomerase I (−7.168 kcal/mol) and with DNA topoisomerase II (–8.542 kcal/mol), and the best values of ligand efficiency (LE was 0.40 and 0.47, respectively).

Based on the data of molecular docking, experiments were carried out on estafiatin **1** and its derivatives on a cell culture of Pliss lymphosarcoma, Walker’s carcinosarcoma, sarcoma 45, alveolar liver cancer PC-1, sarcoma 37, sarcoma 180, and leukemia P-388 and L-1210.

The IC_50_ index was used as a quantitative criterion for the cytotoxicity of the tested compounds (Table 6). In the study of estafiatin **1** and its derivatives on the cell line of Pliss lymphosarcoma, compound **4** turned out to be the most active (IC_50_ = 0.04 ± 0.01 µM). 

Compounds **1** (IC_50_ = 2.13 ± 0.94 µM) and **5** (IC_50_ = 1.34 ± 0.12 µM) were relatively active on the Walker’s carcinosarcoma cell line.

Approximately the same effect on the sarcoma 45 cell line was shown by compounds **13** and **15** with IC_50_ values of 2.68 ± 0.92 µM and 1.84 ± 0.18 µM, respectively. 

On the cell line of alveolar liver cancer PC-1, active compounds **11** and **13** with IC_50_ of 2.96 ± 0.51 µM and 2.80 ± 0.38 µM, respectively, should be noted.

In the study on the cell line of sarcoma 37, compound **11** was relatively active with a value of 0.03 ± 0.01 µM, while compounds **2** and **15** were less active with IC_50_ values of 2.20 ± 0.09 µM and 2.96 ± 1.15 µM, respectively.

In the experiment on the sarcoma 180 cell line, compound **5** was the most active with an IC_50_ value of 0.04 ± 0.01 µM, while compounds **3**, **4**, **6**, and **11** were less active, with an IC_50_ from 2.55 ± 0.90 µM to 2.96 ± 1.07 µM.

On the P-388 leukemia cell line, compounds **1**, **2**, **3**, **11**, and **13** showed higher activity compared to substance **4** (IC_50_ 17.94 ± 2.57 µM).

According to the results of experiments on the leukemia L-1210 cell line, relatively active compounds were **1**, **3**, and **4** with IC_50_ 2.92 ± 1.22 µM, 2.42 ± 0.91 µM, and 1.98 ± 0.06 µM, respectively.

On the cell line of sarcoma 45 to 5-fluorouracil, relatively active compounds were **11** (1.30 ± 0.11 µM) and **13** (1.30 ± 0.08 µM). 

The obtained results demonstrate that a decrease in the viability of the cell line with an increase in the exposure time of estafiatin **1** and its derivatives may indicate the realization of a cytotoxic effect through the induction of apoptosis.

The study of the antitumor activity of estafiatin **1** and its derivatives on six transplanted tumor strains and two types of leukemia P-388 and L-1210, showed that the transformation of the epoxy cycle in the structure of estafiatin **1** into the keto group increased the inhibitory effect of keto derivative **5** against Pliss lymphosarcoma and sarcoma-180 by three to four times compared to the activity of the initial estafiatin **1**. The opening of the epoxy cycle of estafiatin **1** led to the formation of a hydroxyl group at the C-3 position and an exomethylene group at C-4, thereby increasing the antitumor activity against Pliss lymphosarcoma by four times. In the presence of a conjugated 3-keto-4-methylene fragment in the molecule, the activity of such derivative **3** against sarcoma-180, leukemia P-388 and L-1210, and sarcoma-45 resistant to 5-fluorouracil increased by three to six times compared to the effect of estafiatin **1** (Table 7).

From the group of estafiatin derivatives, a relatively high antitumor effect of 3-keto-1,5,7α,6β(H)-guai-4(15),10(14),11(13)-trien-6,12-olide **3**, 3α-acetoxy-1,5,7α,6β(H)-guai-4(15),10(14),11(13)-trien-6,12-olide **4**, and 3-keto-10α(14)-epoxy-1,5,7α,4,6β(H)-guai-11(13)-en-6,12-olide **11** could be observed, which inhibited the growth of Pliss lymphosarcoma, Walker’s carcinosarcoma, sarcoma 45, PC-1 alveolar liver cancer, and sarcoma 180 by 68–90%, and P-388 lymphocytic leukemia and L-1210 lymphoid leukemia (increased expectation of life by 62–104%).

The aforesaid experimental results indicated that the studied samples of estafiatin **1** and its derivatives affect the cellular redox status, forming reactive oxygen species (ROS), thereby causing oxidative damage in the cell and triggering the mitochondrial-dependent pathway of apoptosis [54]. 

## 4. Materials and Methods

### 4.1. Experimental Part

Estafiatin **1** used for the reaction was isolated from the aerial part of the plant *Achillea nobilis* L. according to the previously described method [4]. Column chromatography was carried out on KSK silica gel, the ratio of substance to sorbent = 1:20, and flash chromatography on Armsorbsil 100/160 silica gel. The progress of the reaction and the purity of the derivatives obtained were monitored by TLC. For TLC, Silufol plates TLCP-AF-A-UF of “Imid” company (Krasnodar, Russia) were used, and development was performed by spraying with 1% solution of vanillin H_2_SO_4_, and in a saturated solution of KMnO_4_.

Melting points were determined using an “OptiMelt MPA100” apparatus of Stanford Research Systems company in automatic mode (Sunnyvale, California state, USA). The IR spectrum was recorded on an “Avatar 360 ESP” apparatus of Thermo Nicolet company (Madison, WI, USA) in KBr pellets. Specific optical rotation values were measured on a “Polax-2L” semi-automatic polarimeter of an Atago Co., Ltd company (Tokyo, Japan) in a tube 0.5 dm in length and 3 mL in volume. 

The elemental compositions of the compounds were determined by the combustion method using calculations based on the exact value of the mass numbers of molecular ions, which were determined by high-resolution mass spectrometry on a “Finnigan MAT 8200” instrument (San Jose, CA, USA) (direct input, 120 °C, 70 eV). The same device recorded the mass spectra of the compounds under study. The elemental analysis data of the samples of the compounds were in agreement with the calculated ones.

NMR-spectra were recorded on a “Jeol Resonance-500” spectrometer (Tokyo, Japan) (operating frequency—500.16 MHz for ^1^H, 127.76 MHz for ^13^C, and 121.5 MHz for ^31^P) using “Delta” software for registration of 2D spectra COSY ^1^H–^1^H and ^13^C–^1^H (7 Hz).

The X-ray diffraction experiment was carried out on Bruker P4 (Karlsruhe, Germany) and Syntex P21 (CA, USA) diffractometers (graphite monochromator, λ(Mo–Kα) = 0.71073 Å, room temperature, θ/2θ scanning) for compounds (**33**) and (**34**), respectively. Absorption metering for compound **34** was carried out using experimental azimuthal scanning curves (T_min_/T_max_ = 0.925/0.981) and crystal facet (T_min_/T_max_ = 0.311/0.656) for compound **34**. The structures were deciphered by a direct method. The positions and temperature parameters of non-hydrogen atoms were refined in the isotropic and then in the anisotropic approximation by full-matrix OLS. Hydrogen atoms were placed in geometrically calculated positions and included in the refinement in the “rider” model. All calculations were performed using the Shelx-97 software package, and geometric analysis using Platon software.

The reagents used in the work were purchased from Sigma-Aldrich (St. Louis, MO, USA).

Cytotoxicity of samples in *in vitro* experiments was carried out in collaboration with the Center for Cancer Research of the National Cancer Institute (Bethesda, Rockville, MD, USA).

The antitumor activity of the samples in *in vivo* experiments was carried out jointly with the Laboratory of Experimental Chemotherapy of the Kazakh Research Institute of Oncology and Radiology (Alma-Ata, Republic of Kazakhstan). 

### 4.2. 3,4α-Epoxy-1,5,7α,6β(H)-guai-10(14),11(13)-dien-6,12-olide (**1**)

Isolation of sesquiterpene lactones from the aerial part of *Achillea nobilis* L. and separation and purification of estafiatin (**1**) were carried out according to the method described in [4]. The sum of extractive substances was separated on a column with KSK silica gel at a sum:carrier ratio of 1:10. Elution with benzene gave colorless crystals of the composition C_15_H_18_O_3_, m.p. 104–106 °C, R*_f_* 0.5 (benzene:ethanol 9:1). The yield—0.02%. The spectral data were consistent with the literature [5].

### 4.3. 3α-Hydroxy-1,5,7α,6β(H)-guai-4(15),10(14),11(13)-trien-6,12-olide (**2**)

To a solution of 100 mg (0.4 mmol) of estafiatin (**1**) in 20 mL of toluene at room temperature was added 400 mg (2 mol) of aluminum isopropoxide; the mixture was boiled for 18 h in an argon atmosphere. Next, the solvent was distilled off under pressure; the residue was diluted with 10 mL of ethyl acetate, and 10 mL of 2M HCI solution was added and then stirred for 10 min; then the ethyl acetate layer was separated from the aqueous layer, dried over anhydrous MgSO_4_, filtered off, the solvent was evaporated, and the residue (150 mg) chromatographed on a column with 2 g of silica gel.

When the column was eluted with a mixture of hexane and diethyl ether (2:3), a colorless crystalline substance (**2**) with m.p. 142–144 °C was isolated (diethyl ether), R*_f_* 0.60 (hexane:ether, 2:3). Yield 90 mg (90%). IR spectrum (v_max_, cm^−1^): 3500, 3000, 2950, 2870, 1780, 1680, 1655, 1460, 1430, 1400, 1270, 1160, 1000, 900. Found, %: C 72.97; H 7.21. C_15_H_18_O_3_. Calculated, %: C 73.17; H 7.31.

### 4.4. 3-Keto-1,5,7α,6β(H)-guai-4(15),10(14),11(13)-trien-6,12-olide (**3**)

To a mixture of 13 mL of CHCl_3_ and 2.5 mL (40 mmol) of pyridine, stirring at room temperature, 2 g (20 mmol) of CrO_3_ was added. Then a solution of 250 mg (1.02 mmol) of derivative (**2**) in 1.5 mL of CHCl_3_ was added to this mixture and stirred for 10 min. Then the mixture was filtered, the filtrate was washed with a saturated NaHCO_3_ solution, a 2 M HCl solution, a saturated NaCl solution, dried over anhydrous MgSO_4_, filtered off, the solvent was evaporated, the residue (350 mg) was recrystallized from diethyl ether, and a colorless crystalline substance (**3**) was obtained with m.p. 134–135 °C (diethyl ether), R*_f_* 0.67 (hexane:ether, 2:3). Yield 220 mg (95%). UV spectrum (λ_max_, nm): 217 (ε 14,200). IR spectrum (v_max_, cm^−1^): 3000, 2950, 2870, 1785, 1750, 1680, 1660, 1450, 1420, 1300, 1280, 1190, 1160, 1010, 980, 900. Mass spectrum, *m/z* (I_rel_, %): 244 (M^+^, 41.6), 226(4.2), 215(27.7), 202(5), 186(10.4), 173(11), 159(9.7), 150(100), 145(11.8), 134(18.3), 117(11.1), 105(12.5), 94(9.7), 91(30.5), 79(18), 67(11.1), 53(27), 44(8.3). Found, %: C 73.38; H 6.52. C_15_H_16_O_3_. Calculated, %: C 73.77; H 6.56.

### 4.5. 3α-Acetoxy-1,5,7α,6β(H)-guai-4(15),10(14),11(13)-trien-6,12-olide (**4**)

To a solution of 100 mg of substance (**2**) in 1 mL of pyridine, 2 mL of acetic anhydride was added. The reaction was carried out at room temperature for 20 min. The progress of the reaction was monitored by thin layer chromatography. Next, the reaction mixture was transferred to a separatory funnel, 15 mL of chloroform and a 3% aqueous solution of hydrochloric acid were added, the treatment was repeated 3 times (until neutral), then the organic layer was dried over Na_2_SO_4_, and after 1 h it was filtered. The filtrate was distilled off on a rotary evaporator, an oily substance was formed with composition (**4**), [α]^26^_D_ + 8.8° (*c* 0.005; chloroform), R*_f_* 0.56 (hexane:ether, 1:4). Yield 111 mg (96%). IR spectrum (v_max_, cm^−1^): 3000, 2950, 2880, 1780, 1745, 1680, 1660, 1460, 1420, 1380, 1320, 1250, 1160, 1010, 960, 920, 820. Found, %: C 69.98; H 6.42. C_17_H_20_O_4_. Calculated, %: C 70.83; H 6.94.

### 4.6. 3-Keto-1,5,7α,4,6β(H)-guai-10(14),11(13)-dien-6,12-olide (**5**)

To a solution of 500 mg (2 mmol) of estafiatin (**1**) in 4 mL of CHCl_3_, 0.25 mL of boron trifluoride etherate was added at 0 °C in an argon atmosphere. The mixture was stirred for 5 min, washed with water (3 × 10 mL), dried over anhydrous MgSO_4_, filtered off and the solvent was evaporated, the residue (540 mg) was recrystallized from ethyl acetate, and a colorless crystalline substance (**5**) of the composition C_15_H_18_O_3_ was obtained, m.p. 140–142 °C (from ethyl acetate), [α]^19^_D_ + 126° (*C* 0.005; chloroform), R*_f_* 0.72 (diethyl ether). Yield 450 mg (90%). IR spectrum (v_max_, cm^−1^): 3000, 2950, 2910, 2890, 1780, 1745, 1680, 1655, 1460, 1420, 1340, 1310, 1280, 1260, 1160, 1120, 1100, 1010, 970, 920. Found, %: C 72.97; H 7.21. C_15_H_18_O_3_. Calculated, %: C 73.17; H 7.31.

### 4.7. 3β-Chloro-4α-hydroxy-1,5,7α,6β(H)-guai-10(14),11(13)-dien-6,12-olide (6) and 3α-hydroxy-4β-chloro-1,5,7α,6β(H)-guai-10(14),11(13)-dien-6,12-olide (**7**)

Gaseous hydrogen chloride was passed through a methanol solution of substance 1 (500 mg) for 2–5 min at room temperature. After evaporation of methanol, the mixture was diluted with diethyl ether, then washed successively with 3% sodium bicarbonate solution and water, dried over magnesium sulfate, filtered, and the solvent was distilled off. After treatment and chromatographic purification by elution of the column with a mixture of hexane:ether (3:7), a colorless crystalline substance (**6**) of the composition C_15_H_19_O_3_Cl was isolated, m.p. 205 °C (diethyl ether), [α]^25^_D_ -14° (*C* 0.013; chloroform), R*_f_* 0.55 (diethyl ether). Yield 459.3 mg (80%). IR spectrum (v_max_, cm^−1^): 3535, 3000, 2950, 2880, 1760, 1680, 1660, 1460, 1420, 1340, 1320, 1290, 1280, 1180, 1100, 1000, 980, 940, 910, 820, 740, 710, 680, 610, 510. Mass spectrum, *m/z* (I_rel_, %): 282 (M^+^,29), 264(25), 247(26), 229(39), 91(44), 53(41), 43(100). Found, %: C 62.49; H 6.32; Cl 12.51. C_15_H_19_O_3_Cl. Calculated, %: C 63.72; H 6.73; Cl 12.57.

Elution of the column with a hexane and ether mixture (1:4) yielded a colorless crystalline substance (**7**) with the composition C_15_H_19_O_3_Cl, m.p. 163–166 °C (diethyl ether), [α]^20^_D_ -18° (*c* 0.005; chloroform), R*_f_* 0.45 (diethyl ether). Yield 81.1 mg (15%). IR spectrum (v_max_, cm^−1^): 3536, 3000, 2950, 2880, 1760, 1680, 1659, 1460, 1420, 1340, 1320, 1290, 1280, 1180, 1100, 980, 940, 820, 720, 700, 650, 515. Mass spectrum, *m/z* (I_rel_, %): 282 (M^+^), 264 (M^+^–H_2_O, 11.4), 246(31.4), 228(31.4), 228(10), 221(15.7), 203(21.4), 185(5.7), 179(31.4), 161(20), 145(17.1), 131(24.2), 123(17.1), 117(14.2), 105(22.8), 91(34.2), 85(25.7), 74(10), 67(15.7), 53(32.8), 43(100). Found, %: C 62.58; H 6.26; Cl 12.49. C_15_H_19_O_3_Cl. Calculated, %: C 63.72; H 6.73; Cl 12.57.

### 4.8. 3α-Acetoxy-4β-chloro-1,5,7α,6β(H)-guai-10(14),11(13)-dien-6,12-olide (**8**)

Compound **8** was obtained by interaction (**7**) with acetic anhydride in the manner described in Section 4.5. After treatment and chromatographic purification, a colorless crystalline substance (**8**) with the composition C_17_H_21_O_4_Cl was isolated, m.p. 147–149 °C (diethyl ether), [α]^16.5^_D_ − 55.2° (*C* 0.003; chloroform), R*_f_* 0.64 (diethyl ether). Yield 68.8 mg (60%). IR spectrum (v_max_, cm^−1^): 3000, 2950, 2880, 1772, 1756, 1678, 1656, 1420, 1340, 1320, 1290, 1250, 1180, 1100, 980, 940, 820, 720, 690, 640, 515, 400. Found, %: C 62.43; H 6.36; Cl 10.61. C_17_H_21_O_4_Cl. Calculated, %: C 62.87; H 6.47; Cl 10.94.

### 4.9. General Procedure for the Preparation of Vicinal Diamides (**9**) and (**10**)

First, 200 mg (0.8 mmol) of estafiatin (**1**) was dissolved in 0.4 mL (7.2 mmol) of acetonitrile and benzonitrile, and then 1 mL of sulfuric acid was added dropwise at a temperature of 0 °C. The mixture was kept at this temperature for 25 min. Then water was added and neutralized with sodium carbonate. Extraction with ethyl acetate was conducted followed by drying over sodium sulfate. 

#### 4.9.1. 3,4-Diacetamide-1,5,7α,6β(H)-guai-10(14),11(13)-dien-6,12-olide (**9**)

The residue (0.19 mg) was chromatographed on column with 4 g of silica gel. Elution of the column with benzene isolated a crystalline substance (**9**), m.p. 86–88 °C (benzene), R*_f_* 0.3 (ethyl acetate-benzene 2:3). Yield 145 mg (53%). [α]^20^_D_ + 19° (*C* 0.001; chloroform). IR spectrum (v_max_, cm^−1^): 3520, 3460, 2940, 1760, 1710, 1675, 1635, 1595, 1465, 1310, 1270, 1160, 1120, 1010, 960, 895, 830. Found, %: C 65.86; H 7.55; N 8.11. C_19_H_26_O_4_N_2_. Calculated, %: C 65.89; H 7.51; N 8.09.

#### 4.9.2. 3,4-Dibenzamide-1,5,7α,6β(H)-guai-10(14),11(13)-dien-6,12-olide (**10**)

The residue (0.35 mg) was chromatographed on column with 8 g of silica gel. Elution of the column with a benzene and ethyl acetate mixture (9:1) isolated compound (**10**), m.p. 108–110 °C (from ethanol), R*_f_* 0.37 (ethyl acetate:petroleum ether = 3:2). Yield 210 mg (56%). [α]^20^_D_ + 28° (*C* 0.001; chloroform). IR spectrum (v_max_, cm^−1^): 3400, 2940, 1770, 1710, 1675, 1640, 1595, 1465, 1310, 1270, 1160, 1120, 1010, 960, 895, 830. Found, %: C 74.00; H 6.35; N 5.91. C_29_H_30_O_4_N_2_. Calculated, %: C 74.04; H 6.38; N 5.96.

### 4.10. Epoxidation Reaction

The reaction was carried out at room temperature with the addition of *m*-chloroperbenzoic acid to a stirred solution of estafiatin **1** or estafiatone **5** in chloroform. After the addition of the peracid, the reaction mixture was stirred for 0.5–2 h, after which it was subjected to the usual treatment. The reaction mixture was treated by diluting with a tenfold amount of diethyl ether, sequential washing in a separating funnel with aqueous solutions of sodium chloride, sodium bicarbonate, drying the solution with magnesium sulfate, and removing the solvent under vacuum. The resulting product was recrystallized to yield derivatives (**11**–**16**).

#### 4.10.1. 3-Keto-10α(14)-epoxy-1,5,7α,4,6β(H)-guai-11(13)-en-6,12-olide (**11**)

Compound **11** was obtained by interaction of (**5**) with 66% *m*-chloroperbenzoic acid. After treatment of the reaction mixture in the usual way and chromatography of the resulting residue on a column with silica gel, a colorless crystalline substance (**11**) of the composition C_15_H_18_O_4_ was isolated from the hexane:ethyl acetate (3:7) fraction, m.p. 168–170 °C (from ethyl acetate), [α]^17^_D_ + 20° (*C* 0.004; chloroform), R*_f_* 0.69 (diethyl ether). Yield 55.3 mg (52%). IR spectrum (v_max_, cm^−1^): 3000, 2950, 2920, 2690, 1770, 1740, 1675, 1460, 1420, 1400, 1350, 1310, 1270, 1170, 1140, 1010, 960. Mass spectrum, *m/z* (I_rel_, %): 262 (M^+^, 7.2), 244(14.8), 216(9), 193(12), 149(51.2), 135(29.7), 105(25), 81(52.3), 69(55.4), 57(60.7), 44(100). Found, %: C 68.65; H 6.85. C_15_H_18_O_4_. Calculated, %: C 68.70; H 6.87.

#### 4.10.2. 3-Keto-10β(14)-epoxy-1,5,7α,4,6β(H)-guai-11(13)-en-6,12-olide (**12**)

Elution with a hexane and ethyl acetate mixture (1:4) yielded a colorless crystalline substance (**12**) with the composition C_15_H_18_O_4_, m.p. 187–189 °C (from ethyl acetate), [α]^18^_D_ + 28° (*C* 0.005; chloroform), R*_f_* 0.55 (ether). Yield 12.9 mg (12.2%). IR spectrum (v_max_, cm^−1^): 3000, 2950, 2920, 2890, 1770, 1740, 1675, 1460, 1420, 1400, 1350, 1310, 1270, 1170, 1140, 1010, 960. Found, %: C 68.66; H 6.84. C_15_H_18_O_4_. Calculated, %: C 68.70; H 6.87.

#### 4.10.3. 10α(14)-Epoxy-1,5,7α,4,6β(H)-guai-11(13)-en-4(3),6(12)-diolide (**13**)

Elution of the column with ethyl acetate isolated a colorless crystalline substance (**13**) with the composition C_15_H_18_O_5_, m.p. 149–152 °C (ethyl acetate), [α]^17^_D_ − 18.5° (*C* 0.001; chloroform), R*_f_* 0.47 (diethyl ether). Yield 13 mg (21.3%). IR spectrum (v_max_, cm^−1^): 3010, 3000, 2950, 2870, 1780, 1750, 1680, 1550, 1450, 1390, 1300, 1270, 1240, 1170, 1140, 1030, 940. Mass spectrum, *m/z* (I_rel_, %): 278 (M^+^, 2), 263(4.1), 234(36), 219(16), 206(36), 192(40), 175(49), 162(32), 146(17.5), 133(23), 105(16), 91(35.1), 69(14), 53(100). Found, %: C 64.70; H 6.43. C_15_H_18_O_5_. Calculated, %: C 64.75; H 6.47.

#### 4.10.4. 10β(14)-Epoxy-1,5,7α,4,6β(H)-guai-11(13)-en-4(3),6(12)-diolide (**14**)

Elution of the column with ethyl acetate isolated a colorless crystalline substance (**14**) with the composition C_15_H_18_O_5_, m.p. 139–141 °C (from ethyl acetate), [α]^18^_D_ − 60° (*C* 0.0015; chloroform), R*_f_* 0.41 (diethyl ether). Yield 5.3 mg (5%). IR spectrum (v_max_, cm^−1^): 3010, 3000, 2950, 2870, 1785, 1747, 1680, 1550, 1450, 1310, 1300, 1270, 1240, 1170, 1165, 1140, 1030, 945. Mass spectrum, *m/z* (I_rel_, %): 278 (M^+^), 234 (M^+^–CO_2_, 62), 206(45), 205(39), 192(35), 188(28), 171(9.5), 163(30), 162(30), 161(30), 149(80), 134(39), 119(38), 105(49), 97(45), 91(71), 81(49), 71(61.6), 69(54.8), 57(100). Found, %: C 64.71; H 6.45. C_15_H_18_O_5_. Calculated, %: C 64.75; H 6.47.

#### 4.10.5. 3α(4),10α(14)-Diepoxy-1,5,7α,6β(H)-guai-11(13)-en-6,12-olide (15) and 3α(4),10β(14)-diepoxy-1,5,7α,6β(H)-guai-11(13)-en-6,12-olide (**16**)

It was obtained in the usual way from (**1**) by interaction with 67% *m*-chloroperbenzoic acid. After treatment and chromatographic separation of the residue by elution of the column with a hexane and ethyl acetate mixture (1:1), a colorless crystalline substance (**15**) with composition C_15_H_18_O_4_ was isolated, m.p. 158–160 °C (from ethyl acetate), [α]^20^_D_ − 80° (*c* 0.01; chloroform). R*_f_* 0.64 (diethyl ether). Yield 61.7 mg (58%). IR spectrum (v_max_, cm^−1^): 3030, 2950, 2875, 1780, 1680, 1460, 1420, 1390, 1340, 1320, 1275, 1230, 1150, 1030, 1010, 980, 950, 935. PMR spectrum (CDCl_3_, δ, ppm): 1.64 (3H, s, CH_3_-4); 2.64 and 2.61 (1H each, q, J = 4.5 Hz, CH_2_-10); 3.36 (1H, br.s., H-3); 4.12 (1H, q, J = 11, 9 Hz, H-6); 5.51 and 6.25 (1H each, d, J = 3 Hz CH_2_-13). Found, %: C 68.64; H 6.54. C_15_H_18_O_4_. Calculated, %: C 68.70; H 6.87. 

Elution of the column with a mixture of hexane and ethyl acetate (2:3) isolated a colorless crystalline substance (**16**) with the composition C_15_H_18_O_4_, m.p. 118–121 °C (from ethyl acetate), R*_f_* 0.60 (diethyl ether). Yield 29.8 mg (28%). IR spectrum (v_max_, cm^−1^): 3030, 2950, 2875, 1780, 1680, 1460, 1420, 1390, 1340, 1320, 1275, 1230, 1150, 1030, 1010, 980, 950, 935. PMR spectrum (CDCl_3_, δ, ppm): 1.58 (3H, s, CH_3_-4), 2.63 and 2.61 (1H each, d, J = 4.5 Hz, CH_2_-10), 3.36 (1H, br. s, H-3), 4.13 (1H, q, J = 11.0, 9.0 Hz, H-6), 5.50 and 6.23 (1H each, d, J = 3.0 Hz, CH_2_-13). Found, %: C 68.65; H 6.55. C_15_H_18_O_4_. Calculated, %: C 68.70; H 6.87.

### 4.11. General Procedure for the Preparation of Phosphorus Derivatives of Estafiatin (**17–20**)

A weighed portion of metallic sodium in a ratio of 1:1.2 to estafiatin (**1**) was dissolved in 3 mL of the corresponding dialkyl phosphite at 0 °C and added with intensive stirring of estafiatin (**1**). After completion of the reaction (15–60 min, control by TLC), the mixture was diluted with EtOAc, washed with water (10 mL), saturated with NaCl solution (5 mL), dried over MgSO_4_, filtered off, and then distilled off on a rotary evaporator and purified by column chromatography. The yield of phosphorus derivatives (**17**–**20**) was 65–94.4%.

#### 4.11.1. 3α(4)-Epoxy-13-dimethylphosphonato-1,5,7α,6β(H)-guai-10(14)-en-6,12-olide (**17**)

Oil with composition of C_17_H_25_O_6_P, yield 569 mg (78.8%), was obtained. IR spectrum (v_max_, cm^−1^): 3399, 2925, 2854, 1764 (C=O γ-lactone), 1640 (C=C), 1454, 1378, 1257, 1149 (C–O–C), 1044, 975, 899, 815. Found, %: C 57.26; H 6.89; P 8.53. Calculated, %: C 57.30; H 7.02; P 8.71.

Some cross-peaks in the 2D spectrum ^1^H–^1^H NMR COSY: H-14/H-1, H-6/H-7, H-6/H-5, H-3/H-2a, H-1/H-2a, H-1/H-2b, H-1/H-5, H-11/H-13b, H-11/H-13a, H-13a/H-13b, H-2b/H-2a, H-9a/H-9b, H-9a/H-8b, H-9b/H-8a, H-9b/H-8b.

Some cross-peaks in the 2D spectrum ^1^H–^31^P NMR: H-13a/P, H-13b/P, H-7/P, H-11/P.

#### 4.11.2. 3α(4)-Epoxy-13-diethylphosphonato-1,5,7α,6β(H)-guai-10(14)-en-6,12-olide (**18**)

Oil with composition of C_19_H_29_O_6_P was obtained. Yield 507 mg (65%). IR spectrum (v_max_, cm^−1^): 3446, 2980, 2923, 2852, 1773 (C=O γ-lactone, intensive), 1638(C=C), 1446, 1393, 1329, 1240, 1184, 1167, 1099 (C–O–C), 1026, 967, 904, 819, 719, 690, 668, 529, 502. Found, %: C 59.32; H 7.47; P 8.05. Calculated, %: C 59.38; H 7.55; P 8.07. 

#### 4.11.3. 3α(4)-Epoxy-13-dipropylphosphonato-1,5,7α,6β(H)-guai-10(14)-en-6,12-olide (**19**)

Oil with composition of C_21_H_33_O_6_P was obtained. Yield 790 mg (94.4%). IR spectrum (v_max_, cm^−1^): 3383, 2919, 2851, 1780 (C=O γ-lactone, intensive), 1733, 1652, 1540, 1456, 1066 (C-O-C), 752, 667. Found, %: C 61.15; H 7.97; P 7.50. Calculated, %: C 61.17; H 8.01; P 7.52.

#### 4.11.4. 3α(4)-Epoxy-13-dibutylphosphonato-1,5,7α,6β(H)-guai-10(14)-en-6,12-olide (**20**)

Oil with composition of C_23_H_37_O_6_P, yield 594 mg (67.1%), was obtained. IR spectrum (v_max_, cm^−1^): 3388, 2918, 1745 (C=O γ-lactone, intensive), 1643, 1539, 1464, 1383, 1260, 1069 (C–O–C), 757. Found, %: C 62.82; H 8.55; P 7.07. Calculated, %: C 62.73; H 8.41; P 7.05.

### 4.12. General Procedure for the Amination Reaction

Estafiatin (**1**) was dissolved in ethyl alcohol, and amines (0.88 mmol of monoethanolamine, 0.48 mmol of 25% methylamine, 1.44 mmol of benzylamine, 0.8 mmol of 33% dimethylamine, 1.46 mmol of diethylamine, 1.4 mmol of morpholine, 1.2 mmol of piperidine, 0.96 mmol of diethanolamine) were added into the mixture. The reaction was carried out at room temperature and with constant stirring for one day. After the completion of the reaction, the alcohol was distilled off on a rotary evaporator. The reaction mixture was extracted with ethyl acetate until neutral and then dried over sodium sulfate; the solvent was evaporated under vacuum.

#### 4.12.1. 3α(4)-Epoxy-13-monoethanolamine-1,5,7α,6β(H)-guai-10(14)-en-6,12-olide (21) and 6α-hydroxy-monoethanolamide of estafiatin (**22**)

The reaction mixture (213 mg) was chromatographed on a column with 4 g of silica gel. Elution of the column with a mixture of hexane and ethyl acetate (4:1) isolated compound (**21**) in the form of colorless crystals with the composition C_17_H_25_O_4_N, R*_f_* 0.50 (ethyl acetate:hexane 3:2), m.p. 135–137 °C (ethyl alcohol), [α]^20^_D_ − 18° (*C* 0.01; ethanol). Yield 50 mg (20%). IR spectrum (v_max_, cm^−1^): 3530, 3450, 2930, 1780, 1640, 1450, 1430, 1380, 1270, 1170, 1130, 1020, 910, 825, 760. PMR spectrum (500 MHz, CDCl_3_, δ, ppm, J/Hz): 1.50 (3H, s, H-15), 3.75 (1H, br.s, H-3), 3.95 (1H, t, J = 10.0, H-6), 2.67 (1H, m, H-13a), 2.67 (1H, m, H-13b), 4.75 (1H, br.s, H-14a), 4.82 (1H, br.s, H-14b), 3.50 (1H, br.s, NH), 2.10 (4H, br.s, (CH_2_)_2_). Found, %: C 66.41; H 8.12; N 4.51. Calculated, %: C 66.45; H 8.14; N 4.56.

Elution of the column with ethyl acetate isolated compound (**22**) with the composition of C_17_H_25_O_4_N, R*_f_* 0.08 (ethyl acetate:hexane 3:2), m.p. 156–158 °C (ethyl alcohol), [α]^20^_D_ − 38° (*C* 0.01; ethanol). Yield 176 mg (65%). IR spectrum (v_max_, cm^−1^): 3530, 3450, 2930, 1660, 1459, 1380, 1195, 1020, 900, 800, 750. PMR spectrum (500 MHz, CDCl_3_, δ, ppm, J/Hz): 1.56 (3H, s, H-15), 3.28 (1H, br.s, H-3), 4.0 (1H, t, J = 10.0, H-6), 5.40 (1H, d, J = 2.5, H-13a), 6.1 (1H, d, J = 2.5, H-13b), 4.78 (1H, br.s, H-14a), 4.90 (1H, br.s, H-14b), 2.14 (1H, br.s, NH), 2.17 (4H, br.s, (CH_2_)_2_). Found, %: C 66.43; H 8.11; N 4.52. Calculated, %: C 66.45; H 8.14; N 4.56.

#### 4.12.2. 3α(4)-Epoxy-13-methylamine-1,5,7α,6β(H)-guai-10(14)-en-6,12-olide (**23**) and 6α-hydroxy-methylamide of estafiatin (**24**)

A colorless crystalline substance (**23**) with composition of C_16_H_23_O_3_N, m.p. 138–140 °C (ethyl acetate:hexane) was obtained. Yield 45 mg (30%). [α]^20^_D_ −34° (*C* 0.015; ethanol), R*_f_* 0.48 (ethyl acetate:benzene 3:2). IR spectrum (v_max_, cm^−1^): 3450, 3000, 2890, 2390, 1780, 1650, 1450, 1350, 1180, 1020, 900, 750, 700. PMR spectrum (500 MHz, CDCl_3_, δ, ppm, J/Hz): 1.56 (3H, s, H-15), 2.90 (1H, br.s, H-3), 3.18 (1H, t, J = 10.0, H-6), 2.50 (1H, m, H-13a), 2.50 (1H, m, H-13b), 4.56 (1H, br.s, H-14a), 4.64 (1H, br.s, H-14b), 2.70 (1H, br.s, NH), 1.90 (3H, s, CH_3_). Found, %: C 69.43; H 8.34; N 5.12. Calculated, %: C 69.31; H 8.30; N 5.05.

Elution of the column with ethyl acetate isolated crystalline methylamide (**24**) with the composition C_16_H_23_O_3_N, R*_f_* 0.15 (ethyl acetate:benzene 3:2), m.p. 176–178 °C (from ethyl alcohol). Yield 59 mg (53%). [α]^20^_D_ −101° (*C* 0.1; ethanol). IR spectrum (v_max_, cm^−1^): 3530, 3450, 3400, 3000, 2850, 2390, 1690, 1460, 1350, 1190, 1160, 1050, 900, 800, 700. PMR spectrum (500 MHz, CDCl_3_, δ, ppm, J/Hz): 1.53 (3H, s, H-15), 2.84 (1H, br.s, H-3), 3.34 (1H, t, J = 10.0, H-6), 5.41 (1H, d, J = 3.0, H-13a), 6.21 (1H, d, J = 3.0, H-13b), 4.53 (1H, br.s, H-14a), 4.45 (1H, br.s, H-14b), 2.52 (1H, br.s, NH), 1.84 (3H, s, CH_3_). Found, %: C 69.29; H 8.28; N 5.01. Calculated, %: C 69.31; H 8.30; N 5.05.

#### 4.12.3. 3α(4)-Epoxy-13-benzylamine-1,5,7α,6β(H)-guai-10(14)-en-6,12-olide (**25)**

A crystalline substance, R*_f_* 0.44 (ethyl acetate:hexane, 3:2), m.p. 88–90 °C (ethyl alcohol), [α]^20^_D_ −122° (*C* 0.05; chloroform) was obtained. Yield 407 mg (96%). IR spectrum (v_max_, cm^−1^): 3450, 2935, 2865, 1780, 1640, 1455, 1390, 1270, 1185, 1170, 1085, 1025, 1010, 915, 830, 750, 715. Found, %: C 74.62; H 7.48; N 3.96. C_22_H_27_O_3_N. Calculated, %: C 74.79; H 7.65; N 3.97. PMR spectrum (500 MHz, CDCl_3_, δ, ppm, J/Hz): 1.53 (3H, s, H-15), 2.84 (1H, br.s, H-3), 3.03 (1H, t, J = 9.0, H-6), 2.53 (1H, m, H-13a), 2.53 (1H, m, H-13b), 4.50 (1H, br.s, H-14a), 4.56 (1H, br.s, H-14b), 3.43 (2H, s, –NCH_2_), 7.09 (5H, br.s, Ph).

#### 4.12.4. 3α(4)-Epoxy-13-dimethylamine-1,5,7α,6β(H)-guai-10(14)-en-6,12-olide (**26**)

A crystalline substance, R*_f_* 0.28 (ethyl acetate:hexane, 3:2), m.p. 76–78 °C (ethanol) was obtained. Yield 103 mg (87%). [α]^20^_D_ −12° (*C* 0.001; chloroform). IR spectrum (v_max_, cm^−1^): 2935, 2870, 2825, 2775, 2380, 1770, 1640, 1470, 1270, 1185, 1010, 910, 830. Found, %: C 70.2; H 8.54; N 4.72. C_17_H_25_O_3_N. Calculated, %: C 70.1; H 8.59; N 4.81. PMR spectrum (500 MHz, CDCl_3_, δ, ppm, J/Hz): 1.56 (3H, s, H-15), 2.90 (1H, br.s, H-3), 3.12 (1H, t, J = 10.0, H-6), 2.37 (1H, dd, J = 8.9, H-13a), 2.65 (1H, dd, J = 8.9, H-13b), 4.53 (1H, d, J = 3.0, H-14a), 4.53 (1H, d, J = 3.0, H-14b), 1.89 s (6H, s, –N(CH_3_)_2_).

#### 4.12.5. 3α(4)-Epoxy-13-diethylamine-1,5,7α,6β(H)-guai-10(14)-en-6,12-olide (**27**)

Colorless crystals of composition C_19_H_29_O_3_N, yield 343 mg (90%) were obtained. M.p. 105–107 °C (ethanol), [α]^20^_D_ −18° (*C* 0.001; chloroform). IR spectrum (v_max_, cm^−1^): 2930, 1790, 1630, 1595, 1460, 1380, 1265, 1170, 1020, 830. PMR spectrum (500 MHz, CDCl_3_, δ, ppm, J/Hz): 1.56 (3H, s, H-15), 3.31 (1H, br.s, H-3), 4.0 (1H, br.t, J = 10.0, H-6), 2.43 (1H, m, H-13a), 2.43 (1H, m, H-13b), 4.87 (1H, br.s, H-14a), 4.87 (1H, br.s, H-14b), 2.18 (4H, m, (CH_2_)_2_), 0.93 (6H, t, J = 9, (CH_3_)_2_). Found, %: C 71.45; H 9.05; N 4.35. Calculated, %: C 71.47; H 9.09; N 4.39.

#### 4.12.6. 3α(4)-Epoxy-13-morpholine-1,5,7α,6β(H)-guai-10(14)-en-6,12-olide (**28**)

A crystalline substance of composition C_19_H_27_O_4_N, R*_f_* 0.32 (ethyl acetate:hexane 3:2) was obtained. M.p. 74–76 °C (ethyl alcohol). Yield 332 mg (85%). [α]^20^_D_ −112° (*C* 0.1; chloroform). IR spectrum (v_max_, cm^−1^): 2945, 1770, 1640, 1460, 1310, 1180, 1125, 1080, 1020, 920, 880, 835. PMR spectrum (500 MHz, CDCl_3_, δ, ppm, J/Hz): 1.59 (3H, s, H-15), 2.89 (1H, br.s, H-3), 3.95 (1H, t, J = 10.0, H-6), 2.01 (1H, m, H-13a), 2.01 (1H, m, H-13b), 4.39 (1H, d, J = 2.5, H-14a), 4.39 (1H, d, J = 2.5, H-14b), 3.37 (8H, br. t, J = 4.0, –N(CH_3_)_2_O). Found, %: C 68.45; H 8.10; N 4.19. Calculated, %: C 68.47; H 8.11; N 4.20.

#### 4.12.7. 3α(4)-Epoxy-13-piperidine-1,5,7α,6β(H)-guai-10(14)-en-6,12-olide (**29**)

Colorless crystals of composition C_20_H_29_O_3_N with m.p. 85–88 °C (ethyl alcohol) were obtained. R*_f_* 0.55 (ethyl acetate:hexane, 3:2). Yield 377 mg (95%). [α]^20^_D_ −115° (*C* 0.1; chloroform). IR spectrum (v_max_, cm^−1^): 2940, 1779, 1640, 1450, 1175, 1015, 915, 830. PMR spectrum (500 MHz, CDCl_3_, δ, ppm, J/Hz): 1.56 (3H, s, H-15), 2.87 (1H, br.s, H-3), 3.95 (1H, t, J = 10.0, H-6), 2.50 (1H, br.d, J = 2.5 H-13a), 2.65 (1H, br.d, J = 2.5 H-13b), 4.53 (1H, d, J = 2.5, H-14a), 4.53 (1H, d, J = 2.5, H-14b), 2.78 (10 H, br.s, –N–(CH_2_)_5_). Found, %: C 72.48; H 8.74; N 4.20. Calculated, %: C 72.51; H 8.76; N 4.23.

#### 4.12.8. 3α(4)-Epoxy-13-diethanolamine-1,5,7α,6β(H)-guai-10(14)-en-6,12-olide (**30**)

The resulting residue of the reaction mixture was recrystallized from ethanol. As a result, substance (**30**) was obtained in the form of colorless needle-shaped crystals, R*_f_* 0.15 (ethyl acetate:hexane, 3:2). m.p. 195–197 °C (ethyl alcohol). Yield 242 mg (85%). [α]^20^_D_ −25° (*C* 0.01; chloroform). IR spectrum (v_max_, cm^−1^): 3400, 2930, 1770, 1635, 1450, 1180, 1050, 915, 830, 770. Found, % C 63.21; H 7.52; N 4.35. C_19_H_25_NO_5_. Calculated, %: C 63.16; H 7.74; N 4.33. PMR spectrum (500 MHz, CDCl_3_, δ, ppm, J/Hz): 1.50 (3H, s, H-15), 2.88 (1H, br.s, H-3), 3.18 (1H, t, J = 10.0, H-6), 3.87 (1H, br.s, H-13a), 3.87(1H, br.s, H-13b), 4.65 (1H, d, J = 2.5, H-14a), 4.65 (1H, d, J = 2.5, H-14b), 3.51 (4H, br.s, (CH_2_)_2_).

### 4.13. 3α(4)-Epoxy-13-cytisinyl-1,5,7α,6β(H)-guai-10(14)-en-6,12-olide (**31**)

Compound **31** was obtained by the interaction of estafiatin (**1**) with cytisine (in a ratio of 1:1.2) for 20 h at room temperature. The resulting oil was dissolved in ethyl acetate; on addition of petroleum ether, a precipitate was formed, which was recrystallized from EtOH; as a result, a white crystalline substance with m.p. 214–217 °C, composition C_26_H_32_N_2_O_4_ was obtained. Yield 250 mg (100%). IR spectrum (KBr, ν_max_, cm^−1^): 2973, 2967, 2945, 2934, 2799 (C–H), 1757 (γ-lactone carbonyl), 1652 (C=C), 1579, 1568 (C=C), 1548, 1462, 1450, 1378, 1329, 1300, 1209 (epoxy cycle), 1191 (C–N), 1075, 1048 (CH_2_), 985, 957, 826, 802 (C–O–C). Found, %: C 71.58; H 7.42; N 6.36. Calculated, %: C 71.56; H 7.34; N 6.42.

^1^H NMR spectrum (500 MHz, CDCl_3_, δ, ppm, J/Hz): 1.93 (1H, m, H-1), 1.79 (1H, m, H-2a), 1.96 (1H, m, H-2b), 3.29 (1H, s, H-3), 1.86 (1H, m, H-5), 3.81 (1H, t, J = 10.0, H-6), 1.93 (1H, m, H-7), 2.20 (1H, ddd, J = 20.0, 9.7, 2.5, H-8a), 2.79 (1H, dd, J = 9.7, 5.7, H-8b), 1.68 (1H, ddd, J = 13.96, 10.52, 6.0, H-9a), 2.00 (1H, dd, J = 13.96, 7.3, H-9b), 2.10 (1H, dd, J = 11.0, 8.5, H-11), 2.47 (1H, dd, J = 13.5, 10.0, H-13a), 2.72 (1H, dd, J = 13.5, 3.5, H-13b), 4.74 (1H, s, H-14a), 4.79 (1H, s, H-14b), 1.50 (3H, s, H-15), 6.44 (1H, dd, J = 9.0, 1.1, H-3′), 7.25 (1H, dd, J = 9.0, 6.8, H-4′), 5.96 (1H, dd, J = 6.8, 1.1, H-5′), 2.95 (1H, br.s, H-7′), 1.74 (1H, m, H-8′a), 1.88 (1H, m, H-8′b), 2.42 (1H, br.s, H-9′), 3.87 (1H, dd, J = 15.0, 10.0, H-10′a), 4.02 (1H, d, J = 15.0, H-10′b), 2.49 (1H, br.d, J = 11.0, H-11′a), 2.86 (1H, br.d, J = 11.0, H-11′b), 2.25 (1H, dd, J = 11.0, 2.0, H-13′a), 2.88 (1H, br.d, J = 11.0, H-13′b). 

^13^C NMR spectrum (125 MHz, CDCl_3_, δ, ppm, J/Hz): 30.59 (d, C-1), 31.12 (t, C-2), 63.27 (d, C-3), 66.04 (s, C-4), 48.01 (d, C-5), 80.81 (d, C-6), 44.40 (d, C-7), 44.54 (t, C-8), 32.77 (t, C-9), 146.84 (s, C-10), 50.40 (d, C-11), 177.04 (s, C-12), 58.58 (t, C-13), 114.35 (q, C-14), 18.79 (q, C-15), 163.35 (s, C-2′), 116.95 (d, C-3′), 138.47 (d, C-4′), 104.56 (d, C-5′), 151.31 (s, C-6′), 35.25 (d, C-7′), 25.78 (t, C-8′), 28.24 (d, C-9′), 50.01 (t, C-10′), 62.47 (t, C-11′), 59.46 (t, C-13′).

### 4.14. 3α(4)-Epoxy-13-anabasinyl-1,5,7α,6β(H)-guai-10(14)-en-6,12-olide (**32**)

Compound **32** was obtained by the interaction of estafiatin (**1**) in a solution of methanol with anabasine (in a ratio of 1:2) for 26 h at room temperature. The resulting oil was dissolved in ethyl acetate; the addition of petroleum ether precipitated a precipitate, which was recrystallized from EtOH; as a result, a white crystalline substance with the composition C_25_H_32_N_2_O_3_ was obtained, m.p. 131.8–134.6 °C (ethyl acetate:hexane). Yield 330 mg (88%.). IR spectrum (KBr, ν_max_, cm^−1^): 2984, 2947, 2932, 2912 (C–H), 1774 (C=O γ-lactone), 1633 (C=N), 1591, 1577, 1568 (C=C), 1453, 1443, 1427, 1327, 1303, 1209 (epoxy cycle), 1199 (C–N), 1054, 1024, 1003 (CH_2_), 985, 961, 826, 802 (C–O–C). Found, %: C 73.51; H 7.86; N 6.85. Calculated, %: C 73.53; H 7.84; N 6.86.

^1^H NMR spectrum (500 MHz, CDCl_3_, δ, ppm, J/Hz): 3.10 (1H, m, H-1), 2.18 (2H, m, H-2a,b), 3.34 (1H, s, H-3), 2.30 (1H, m, H-5), 3.95 (1H, t, J = 10.5, H-6), 2.35 (1H, m, H-7), 2.12 (2H, m, H-8a,b), 2.22 (2H, m, H-9a,b), 1.55 (1H, m, H-11), 2.31 (1H, dd, J = 13.53, 2.72, H-13a), 2.66 (1H, dd, J = 13.53, 6.87, H-13b), 4.87 (1H, s, H-14a), 4.92 (1H, s, H-14b), 1.53 (3H, s, H-15), 1.69 (1H, m, H-2a,b’), 1.72 (1H, m, H-3a,b’), 1.78 (1H, d, J = 1.8, H-4a’), 2.30 (1H, d, J = 1.8, H-4b’), 1.33 (1H, m, H-5a,b’), 2.84 (1H, d, J = 10.5, H-6a’), 2.93 (1H, d, J = 10.0, H-6b’), 8.5 (1H, d, J = 1.8, H-8′), 7.66 (1H, d, J = 7.8, H-10′), 7.25 (1H, dd, J = 7.8, 1.8, H-11), 8.48 (1H, dd, J = 4.8, 1.8, H-12′).

^13^C NMR spectrum (125 MHz, CDCl_3_, δ, ppm, J/Hz): 30.21 (d, C-1), 30.67 (t, C-2), 63.47 (d, C-3), 66.33 (s, C-4), 44.75 (d, C-5), 80.79 (d, C-6), 42.64 (d, C-7), 44.75 (t, C-8), 32.88 (t, C-9), 147.12 (s, C-10), 51.05 (d, C-11), 178.05 (s, C-12), 54.05 (t, C-13), 114.37 (q, C-14), 18.66 (q, C-15), 66.12 (d, C-2′), 36.46 (t, C-3′), 24.56 (t, C-4′), 25.72 (t, C-5′), 51.92 (t, C-6′), 148.76 (d, C-8′), 140.30 (s, C-9′), 135.56 (d, C-10′), 123.65 (d, C-11′), 149.61 (d, C-12′).

#### 4.14.1. 3α(4)-Epoxy-10(14),11(13)-bis-(dicholoro-cyclopropano)-1,5,7α,6β(H)-guai-6,12-olide (**33**)

A solution of 3 mL of CHCl_3_, 2 mL of 50% aqueous NaOH and 30 mg of crown-ether was stirred at room temperature, and 100 mg (0.0004 mol) of estafiatin **1** was added. After the completion of the reaction, the chloroform layer was treated, and the chloroform layer was dried over MgSO_4_. The residue (0.21 g) was chromatographed on a SiO_2_ column (eluent petroleum ether and ethyl acetate with an increase in the concentration of the latter), and 0.052 g (31%) of product (**33**) was isolated with m.p. 194–196 °C (petroleum ether:EtOAc, 3:2), R*_f_* 0.44 (petroleum ether:EtOAc, 3:2). Mass spectrum, *m/z* (I_rel_, %): 410 (M^+^, 2), 397 (9), 327 (13), 199 (6), 115 (14), 95 (34), 66 (16), 65 (16), 43 (100). Found, %: C 49.48; H 4.36; Cl 34.29. C_17_H_18_Cl_4_O_3_. Calculated, %: C 49.51; H 4.37; Cl 34.47. IR spectrum (v_max_, cm^−1^): 3088, 3011, 2978, 2938, 2896, 2869, 1781 (C=O γ-lactone), 1464, 1451, 1433, 1412, 1380, 1351, 1330, 1319, 1301, 1287, 1257, 1217, 1207, 1184, 1152, 1102, 1077, 1055, 1035, 1019, 1000, 986, 961, 954, 933, 920, 897, 875, 867, 828, 815, 777, 757 (C–Cl), 700, 681, 625, 591, 582, 510, 504, 487, 477, 461, 440.

PMR spectrum (500 MHz, CDCl_3_, δ, ppm, J/Hz): 3.34 (s, 1H, H-3), 4.10 (dd, 1H, J = 8.6, H-6), 2.02 (d, 1H, J = 7.0, H-13a), 1.82 (d, 1H, J = 7.0, H-13b), 1.32 (d, 1H, J = 7.0, H-14a), 1.22 (d, 1H, J = 7.0, H-14b), 1.58 (s, 3H, CH_3_-15).

^13^C NMR spectrum (125.75 MHz, CDCl_3_): 40.58 (d, C-1), 30.39 (t, C-2), 42.72 (d, C-3), 37.42 (s, C-4), 60.75 (d, C-5), 80.07 (d, C-6), 48.38 (d, C-7), 25.39 (t, C-8), 29.29 (t, C-9), 35.18 (s, C-10), 31.87 (s, C-11), 171.43 (s, C-12), 26.36 (t, C-13), 65.42 (q, C-14), 18.50 (q, C-15), 61.13 (s, C-16), 67.32 (s, C-17).

Crystallographic data and parameters of the X-ray diffraction experiment for lactone (**33**) were obtained: C_17_H_18_Cl_4_O_3_, M = 411.11, orthorhombic system, spase group P212121, a = 6.4539 (5), b = 15.009 (1), c = 19.453 (2) Å, V = 1884.3 (3) Å3, Z=4, d_cal_ = 1.449 g·cm^−3^, μ = 0.640 mm^−1^, scanning region 2θ < 54°, 2374 of measured reflections, 2077 reflections with *I* ≥ 2σ(*I*), 217 of refined parameters, R_1_[*I* ≥ 2σ(*I*)] = 0.0425, wR_2_ = 0.1228 (over all reflections), absolute structure parameter (Flack) 0.0(1).

#### 4.14.2. 3α(4)-Epoxy-11(13)-dibromo-cyclopropano-1,5,7α,6β(H)-guai-10(14)-en-6,12-olide (**34**)

To 3 mL of bromoform, 0.06 g of crown ether and 50% NaOH solution were added and mixed. At room temperature, 0.2 g (0.4 mmol) of estafiatin **1** was added. Over time, the solution turned brown. After three hours, water was added to the solution and extracted by chloroform. The organic layer was dried over MgSO_4_. After filtration, it was distilled off on a rotary evaporator. The resulting reaction mixture was chromatographed on a silica gel column eluting with petroleum ether and ethyl acetate with an increase in the concentration of the latter. At the same time, colorless crystals with m.p. 204–206 °C (petroleum ether:EtOAc, 1:1), Rf 0.66 (petroleum ether:EtOAc, 1:1) was isolated. Yield 0.035 g (21%). IR spectrum (v_max_, cm^−1^): 1768 (C=O), 1641 (C=C), 682 (C–Br). Found, %: C 45.91; H 4.29; Br 37.09. C_16_H_18_Br_2_O_3_. Calculated, %: C 45.93; H 4.31; Br 38.28.

PMR spectrum (500 MHz, CDCl_3,_ δ, ppm, J/Hz): 1.58 (s, 3H, CH_3_-15), 3.36 (s, 1H, H-3), 4.17 (dd, 1H, J = 8.0; 6.0, H-6), 4.79 (s, 1H, H-14a), 4.94 (s, 1H, H-14b).

Crystallographic data and parameters of the X-ray diffraction experiment for lactone (**34**) were obtained: C_16_H_18_Br_2_O_3_ = 418.12, orthorhombic system, spase group P212121, a = 8.039 (4), b = 11.255 (5), C = 17.546 (8), V = 1588 (1) Å3, Z = 4, d_cal_ = 1.749 g·cm^−3^, μ = 5.113 mm^−1^, scanning region 2θ < 50°, 1620 of measured reflections, 1046 reflections with *I* ≥ 2σ(*I*), 190 of refined parameters, R_1_[*I* ≥ 2σ(*I*)] = 0.0579, wR_2_ = 0.1335 (over all reflections), absolute structure parameter (Flack)—0.05 (5).

### 4.15. Computer Simulation of the Interaction Energy of the “Ligand–Target” Complex

Molecular docking was performed using the Maestro graphical interface of the Schrödinger Suite software package. The SP (standard precision) docking mode was used. As the final results, the value of the scoring function GScore was used, which shows the binding energy of the ligand to the target molecule.

Ligand efficiency (LE) was calculated using the formula (-GScore)/HA, where GScore is the calculated estimated binding energy, and HA is the number of heavy atoms in the ligand structure. Values ≥ 0.3 were taken as an acceptable level of ligand efficacy [55].

### 4.16. Cytotoxicity *in vitro*

The cytotoxicity of the compounds was determined using cell lines of Pliss lyphosarcoma, Walker’s carcinosarcoma, sarcoma 45, sarcoma-180, alveolar liver cancer PC-1, leukemia P-388, leukemia L-1210, and sarcoma 45 resistant to 5-fluorouracil. Tests were performed in 96-well plates (Falcon) with an inoculum of 2.5 × 10^4^ cells/mL. Test solutions were prepared as stock solutions in ethanol. The final ethanol concentration was 1% (*v*/*v*) or less. To quantify cytotoxicity, 15 µL of an aqueous solution of methylthiazolyl tetrazolium chloride (MTT, Fluka, 5 mg/mL in PBS) was added after 72 h. When incubated at 37 °C for 4 h, the surviving cells metabolized MTT into an insoluble formazan dye. The culture medium was removed, and the formazan dye was dissolved using 150 µL of 10% SDS (sodium dodecyl sulfate) in water. After 24 h of incubation at room temperature, absorbance was measured at 540 nm using a microplate reader (MRX, Dynex Technologies). An optical density dependence diagram from logarithmic concentration was plotted to determine IC_50_ values, and eight different concentrations were tested [56]. 

### 4.17. Antitumor Activity

The antitumor activity of the substances was studied in white outbred rats with transplanted tumors of mice and rats. The antitumor effect of the substances was determined with daily intraperitoneal administration in a 2% solution of dimethylsulfoxide (DMSO) for 5 days at the maximum tolerated doses (MTD). To assess the antitumor activity of the substances, we used the percentage of tumor growth inhibition and the magnitude of the increase in average life expectancy, determined immediately after the end of treatment [57]. 

### 4.18. Statistical Processing of Results

Statistical processing of the results was carried out using the program “GraphPad Prism v. 6.0”. The results obtained were presented as “mean value ± standard error of the mean”. Differences were considered significant at the achieved level of significance *p* < 0.05.

## 5. Conclusions

Chemical modification of the guaian-type sesquiterpene lactone estafiatin **1**, which has the structure 3,4α-epoxy-1,5,7α,6β(H)-guai-10(14),11(13)-dien-6,12-olide, allows promising new biologically active derivatives to be obtained. As a result of our chemical modification of molecule **1**, 33 new polyfunctional compounds were synthesized.

When molecule **1** interacts with acidic reagents, the reactions proceed through the C3–C4 epoxy cycle and the C10=C14 exomethylene double bond stereospecifically.

Upon opening of the epoxy ring in molecule **1**, the reaction proceeds regioselectively.

The interaction of estafiatin **1** with primary and secondary amines according to the type of Michael reaction proceeds chemoselectively at the exomethylene group of γ-lactone with the formation of quantitatively 12 new amino derivatives, of which two are hybrid molecules of the initial lactone with the alkaloids cytisine and anabasine.

For the first time, the reactions of phosphorylation and cyclopropanation at the exomethylene group of the γ-lactone of estafiatin **1** were carried out, which proceed regio- and stereospecifically.

When determining cytotoxicity and antitumor activity by molecular docking and *in vitro* and *in vivo* methods of samples **1** and synthesized derivatives, 5 compounds were selected: isozaluzanin C **2**, 3-keto-4-methylene-*cis*-guaianolide **3**, 3α-acetoxy-isozaluzanin C **4**, estafiatone **5,** and 10α(14)-epoxy-1,5,7α,4,6β(H)-guai-11(13)-en-4(3),6(12)-diolide **13**.

According to the results of the experiments, it was found that the introduction of a hydroxy group into molecule **1** at the C-3 position increases the antitumor activity of the synthesized sample **2** against Pliss lymphosarcoma, Walker’s carcinosarcoma, sarcoma 45, alveolar liver cancer PC-1, sarcoma 180, and leukemia P-388. At the same time, 3-keto-4-methylene-cis-guaianolide **3** inhibits the growth of Pliss’s lymphosarcoma, Walker’s carcinosarcoma, sarcoma 45, alveolar liver cancer PC-1, and leukemia P-388 and L-1210, and in the case of estafiatone **5**, Pliss’s lymphosarcoma, Walker’s carcinosarcoma, sarcoma 45, alveolar liver cancer PC-1, and sarcoma 180.

Acetylation of isozaluzanin C **2** and epoxidation at C10=C14 of the double bond in molecule **5** increased the antitumor effect of samples **4** and **13** against 8 types of transplanted tumor strains from two to five times compared with the initial lactone **1**.

Thus, 3,4α-epoxy-1,5,7α,6β(H)-guai-10(14),11(13)-diene-6,12-olide **1** and its derivatives 3-keto-4-methylene-*cis*-guaianolide **3**, 3α-acetoxy-isozaluzanin C **4,** and 10α(14)-epoxy-1,5,7α,4,6β(H)-guai-11(13)-en-4(3),6(12)-diolide **13** are potential sources for the development of new anticancer drugs based on them.

## Figures and Tables

**Table 1:** Chemical shifts (in ppm) and spin–spin coupling constants (^1^H NMR, 500.16 MHz, CDCl_3_, δ, ppm, in brackets, in Hz) for estafiatin **1** and its derivatives **2**–**10**.

No.	1	2	3	4	5	6	7	8	9	10
Me-4	1.53 s	-	-	-	1.22 d (6)	1.34 s	1.51 s	1.74 s	1.18 s	1.21 s
H-3	3.28 br.s	4.68 t (8)		5.62 br.t (6)	-	4.26 q (12.5;7)	3.94 br.d (3)	5.33 d (4.5)	3.75 t	3.70 t
H-4	-	-	-	-	2.50 m	-	-	-	-	-
H-6	4.01 q (10.5:9)	3.90 t (9)	3.99 t (9)	3.87 t (9)	3.95 t (8.5)	4.17 q (11;9)	4.23 q (10.5;9)	4.39 q (10;9)	3.87 t (10)	3.90 t (10;5)
H-13a	5.42 d (3.5)	5.48 d (3.5)	5.56 d (3)	5.47 d (3)	5.52 d (3)	5.58 d (3)	5.48 d (3)	5.51 d (3.5)	5.43 d (2.5)	4.84 d (2.5)
H-13b	6.12 d (3.5)	6.21 d (3.5)	5.85 d (3)	6.18 d (3)	6.22 d (3)	6.26 d (3)	6.20 d (3)	6.22 d (3,5)	6.15 d (2,5)	6.0 d (2,5)
H-14a	4.78 br.s	4.78 br.s	4.67 s	4.76 br.s	4.62 br.s	5.01 br.s	4.94 br.s	5.08 br.s	5.93 br.s	5.71 br.s
H-14b	4.78 br.s	4.90 br.s	4.91 s	4.90 br.s	4.92 br.s	5.08 br.s	4.94 br.s	5.08 br.s	5.93 br.s	5.71 br.s
H-15a	-	5.35 br.s	6.23 d (2)	5.36 br.s	-	-	-	-	-	-
H-15b	-	5.45 br.s	6.28 d (2)	5.51 br.s	-	-	-	-	-	-
Other protons	-	-	-	–OAc 2.02 s	-	-	-	–OAc 2.06 s	(–NH–C(O)CH_3_)_2_ 1.23 s, (6H)	Aromatic ring 7.06 br.s (10H)

**Table 2:** Chemical shifts (in ppm) and spin–spin coupling constants (^1^H NMR, 500.16 MHz, CDCl_3_, δ, ppm, in brackets, in Hz) for estafiatin derivatives **11**–**14**.

No.	Me-4	H-4	H-6	H-13a	H-13b	H-14a	H-14b
**11**	1.24 d (6.5)	2.35 m	4.41 t (9.5)	5.58 d (3.5)	6.29 d (3.5)	2.42 d (4)	2.61 d (4)
**12**	1.19 d (7)	2.25 m	4.36 t (10)	5.52 d (3)	6.24 d (3)	2.51 d (4.5)	2.73 d (4.5)
**13**	1.50 d (6,5)	4.78 q (12.5;6.5)	4.17 q (10.5;9)	5.76 d (3)	6.31 d (3)	2.76 d (4.5)	2.82 d (4.5)
**14**	1.32 d (6.5)	4.72 q (12.5;6.5)	4.36 t (9.5)	5.39 d (3)	6.20 d (3)	2.56 d (4.5)	2.78 d (4.5)

**Table 3:** ^1^H NMR spectra data for derivatives **17**–**20** (500.16 MHz, CDCl_3_, δ, ppm, J/Hz).

H Atom	17	18	19	20
1	2	3	4	5
1	2.87 (ddd, J = 10.5, 9.0, 1.0)	2.88 (ddd, J = 10.5, 8.0, 1.0)	2.87 (ddd, J = 9.0, 10.5, 1.0)	2.87 (ddd, J = 9.0, 10.5, 1.0)
2a	1.76 (ddd, J = 14.0, 10.5, 1.0)	1.76 (ddd, J = 13.7, 10.5, 1.0)	1.76 (ddd, J = 13.7, 10.5, 1.0)	1.76 (ddd, J = 13.7, 10.5, 1.0)
2b	2.03–2.10 m	2.04–2.09 m	2.03–2.13 m	2.03–2.13 m
3	3.33 (d, J = 1.0)	3.34 (d, J = 1.0)	3.33 (d, J = 1.0)	3.37 (d, J = 1.0)
5	2.27–2.29 m	2.3–2.37 m	2.32–2.36 m	2.31–2.35 m
6	3.99 (dd, J = 10.5, 9.0)	4.0 (t, J = 10.5, 9.5)	3.99 (dd, J = 10.5, 9.5)	3.99 (dd, J = 10.6, 9.6)
7	2.21–2.26 m	2.23–2.28 m	2.16–2.25 m	2.15–2.25 m
8a	2.21–2.26 m	2.17–2.25 m	2.22–2.27 m	2.22–2.27 m
8b	2.03–2.13 m	2.08–2.15 m	2.03–2.13 m	2.03–2.13 m
9a	1.36 m	1.36 m	1.35 m	1.31–1.41 m
9b	2.21–2.26 m	2.17–2.26 m	2.16–2.25 m	2.15–2.25 m
11	2.53 (dddd, J = 12.0, 7.5, 5.0, J_PH_ = 20.5)	2.53 (dddd, = 12.0, 7.2, 4.5, J_PH_ = 21.5)	2.52 (dddd, J = 11.8, 7.4, 4.4, J_PH_ = 22.1)	2.52 (dddd, = 11.8, 7.4, 4.7, J_PH_ = 22.0)
13a	2.34 (ddd, J = 16.2, 5.0, J_PH_ = 19. 5)	2.34 (ddd, J = 15.7, 4.5, J_PH_ = 20.0)	2.34 (ddd, J = 15.7, 4.5, J_PH_ = 20.0)	2.33 (ddd, J = 16.2, 4.7, J_PH_ = 19.4)
13b	1.90 (ddd, J = 16.2, 7.5, J_PH_ = 18.4)	1.90 (ddd, J = 15.7, 7.2, J_PH_ = 18. 0)	1.97 (ddd, J = 15.7, 7.4, J_PH_ = 18.3)	1,92 (ddd, J = 16.2, 7.4, J_PH_ = 18.0)
14a	4.85 (t, J = 1.0)	4.86 s	4.84 (t, J = 1.0)	4.89 (t, J = 1.0)
14b	4.78 (d, J = 1.0)	4.80 s	4.79 (d, J = 1.0)	4.84 (d, J = 1.0)
15	1.54 (3H, s)	1.55 (3H, s)	1.54 (3H, s)	1.59 (3H, s)
1’	3.73 (3H, d, J_PH_ = 5.0)	4.09 (4H, m)	3.96 (4H, m)	4.0 (4H, m)
1”	3.71 (3H, d, J_PH_ = 5.0)		1.66 (4H, m)	1.66 (4H, m)
2’	-	1.3 (6H, t, J = 9.3)		
3’	-	-	0.93 (6H, t, J = 7.5)	1.36 (4H, m)
4’	-	-	-	0.95 (6H, t, J = 7.5)

**Table 4:** ^13^C NMR spectra data for derivatives **17**–**20** (127.76 MHz, CDCl_3_, δ, ppm) (P, J/Hz).

C Atom	17	18	19	20
1	2	3	4	5
1	44.2 d	44.2 d	44.2 d	44.2 d
2	32.6 t	32.6 t	32.6 t	32.6 t
3	63.0 d	63.1 d	63.0 d	63.0 d
4	65.8 s	65.8 s	65.8 s	65.8 s
5	50.3 d	50.4 d	50.3 d	50.3 d
6	80.9 d	80.8 d	80.8 d	80.8 d
7	47.7 d (d, J = 4.0)	47.6 d (d, J = 3.7)	47.7 d (d, J = 3.4)	47.7 d (d, J = 4.7)
8	30.2 t	30.3 t	30.4 t	30.4 t
9	30.5 t	30.7 t	30.7 t	30.7 t
10	146.7 s	146.7 s	146.7 s	146.8 s
11	41.8 d (d, J = 4.2)	42.0 d (d, J = 4.3)	42.0 d (d, J = 4.3)	42.0 d (d, J = 4.3)
12	176.4 s (d, J = 13.8)	176.6 s (d, J = 13.1)	176.5 s (d, J = 13.9)	176.5 s (d, J = 13.8)
13	24.1 t (d, J = 145.5)	25.1 t (d, J = 145.2)	25.0 t (d, J = 145.2)	25.0 t (d, J = 143.6)
14	114.2 t	114.2 t	114.2 t	114.1 t
15	18.5 q	18.6 q	18.6 q	18.6 q
1’	52.7 q (d, J = 6.7)	62.1 t (d, J = 6.7)	67.5 t (d, J = 6.7)	65.8 t (d, J = 6.7)
1”	52.2 q (d, J = 6.7)	61.7 t (d, J = 6.7)	67.1 t (d, J = 6.7)	65.41 t (d, = 6.7)
2’	-	16.3 q	23.7 t	32.4 t
2”	-	16.2 q	23.7 t	32.3 t
3’	-	-	10.0 q	18.6 t
3”	-	-	10.0 q	18.6 t
4’	-	-	-	13.5 q
4”	-	-	-	13.5 q

**Table 5:** Ligand efficiency and values of the estimated binding energy of the complexes of the studied compounds with the receptors DNA topoisomerase I and DNA topoisomerase II.

Compound	Receptor DNA Topoisomerase I		Receptor DNA Topoisomerase II	
	Estimated Binding Energy, kcal/mol	Ligand Efficiency	Estimated Binding Energy, kcal/mol	Ligand Efficiency
**1**	−6.439	0.36	−7.721	0.43
**2**	−7.168	0.40	−8.542	0.47
**3**	−6.643	0.37	−8.013	0.45
**4**	−7.041	0.34	−6.802	0.32
**5**	−6.494	0.36	−8.286	0.46
**6**	−7.013	0.37	−7.951	0.42
**7**	−6.132	0.28	−7.721	0.41
**8**	−6.485	0.34	−5.827	0.26
**11**	−6.421	0.34	−7.592	0.40
**12**	−6.368	0.34	−7.659	0.40
**13**	−6.261	0.31	−8.458	0.42
**14**	−6.057	0.30	−7.941	0.40
**15**	−6.224	0.33	−7.763	0.41
**16**	−6.106	0.32	−7.429	0.39
**17**	−5.865	0.24	−6.384	0.25
**18**	−5.616	0.22	−7.397	0.31
**19**	−5.733	0.20	−5.013	0.18
**20**	−5.609	0.19	−5.992	0.20
**33**	−5.754	0.24	−3.833	0.16
**34**	−4.625	0.22	−6.859	0.33

**Table 6:** IC_50_ antitumor activity of estafiatin **1** and its derivatives.

Compound	1	2	3	4	5	6	11	13	15
Pliss lymphosarcoma (IC_50_), µM	4.62 ± 1.42	4.65 ± 1.01	2.68 ± 0.92	0.04 ± 0.01	4.34 ± 1.73	5.09 ± 1.70	4.82 ± 1.35	1.84 ± 0.18	4.19 ± 1.03
Walker’s carcinosarcoma (IC_50_), µM	2.13 ± 0.94	4.27 ± 1.58	5.16 ± 1.52	4.93 ± 0.92	1.34 ± 0.12	5.51 ± 1.47	+infinity	8.37 ± 1.92	3.00 ± 1.42
Sarcoma 45 (IC_50_), µM	4.37 ± 1.26	3.69 ± 1.05	3.34 ± 0.98	2.96 ± 0.75	3.86 ± 1.79	3.97 ± 1.04	4.69 ± 1.42	2.59 ± 0.75	2.00 ± 0.98
PC-1 alveolar liver cancer (IC_50_), µM	3.05 ± 1.67	5.09 ± 1.75	3.05 ± 0.86	4.69 ± 1.01	3.05 ± 0.90	3.61 ± 1.75	2.96 ± 0.51	2.80 ± 0.38	3.17 ± 1.04
Sarcoma 37 (IC_50_), µM	3.86 ± 0.97	2.20 ± 0.09	4.34 ± 1.45	3.05 ± 1.08	2.96 ± 0.07	3.05 ± 1.05	0.03 ± 0.01	3.17 ± 1.22	2.96 ± 1.15
Sarcoma 180 (IC_50_), µM	3.05 ± 1.10	4.52 ± 1.42	2.96 ± 1.01	2.96 ± 1.07	0.04 ± 0.01	2.96 ± 0.87	2.55 ± 0.90	4.52 ± 1.68	3.61 ± 1.47
Leukemia P-388 (IC_50_), µM	3.62 ± 1.21	2.92 ± 1.09	2.96 ± 0.95	17.94 ± 2.57	-	-	-	1.69 ± 0.15	4.52 ± 1.25
Leukemia L-1210 (IC_50_), µM	2.92 ± 1.22	-	2.42 ± 0.91	1.98 ± 0.06	-	-	-	4.52 ± 1.59	2.79 ± 0.69
Sarcoma 45 to 5-fluoro-uracil (IC50), µM	3.05 ± 1.47	-	2.29 ± 0.72	5.09 ± 2.03	-	-	-	1.30 ± 0.11	1.30 ± 0.08

- reliability of differences *p* < 0.05 compared with the comparison group.

**Table 7:** Antitumor activity on transplanted tumor strains of estafiatin **1** and its derivatives.

Compound	Dose mg/kg	Pliss Lymphosarcoma	Walker’s Carcinosarcoma	Sarcoma 45	PC-1 Alveolar Liver Cancer	Sarcoma 37	Sarcoma 180	Leukemia P-388	Leukemia L-1210	Sarcoma 45 to 5-Fluoro- Uracil
**1**	20	23.3 ± 1.45	59.9 ± 1.04	31.2 ± 0.32	55.0 ± 1.77	15.1 ± 0.14	26.4 ± 0.17	16.1 ± 0.02	12.4 ± 1.12	21.3 ± 0.19
**2**	20	80.0 ± 1.04	55.9 ± 1.40	51.2 ± 1.21	63.0 ± 1.60	25.3 ± 1.72	41.3 ± 1.41	96.0 ± 2.75	-	-
**3**	20	68. 7 ± 0.94	69.1 ± 1.26	68.9 ± 1.10	68.2 ± 1.33	29.6 ± 1.50	81.5 ± 1.72	81.5 ± 2.07	45.0 ± 1.06	88.9 ± 1.64
**4**	30	73.1 ± 1.83	64.0 ± 1.42	52.0 ± 1.92	70.4 ± 0.15	84.0 ± 2.43	55.5 ± 2.01	79.4 ± 1.92	23.1 ± 0.05	76.2 ± 2.77
**5**	25	71.1 ± 1.93	59.0 ± 1.61	48.1 ± 0.47	79.0 ± 0.09	36.0 ± 1.05	74.1 ± 2.32	-	-	-
**6**	25	81.4 ± 1.45	67.9 ± 0.75	50.1 ± 1.67	56.8 ± 2.45	32.0 ± 1.97	50.0 ± 0.99	-	-	-
**11**	30	41.1 ± 0.83	51.3 ± 1.32	21.4 ± 0.05	70.0 ± 1.90	42.1 ± 2.00	61.0 ± 1.67	-	-	-
**13**	25	68.9 ± 0.71	83.0 ± 1.53	90.9 ± 1.01	43.6 ± 2.01	49.4 ± 1.47	72.1 ± 2.04	104.0 ± 2.90	62.0 ± 1.70	75.7 ± 2.70
**15**	25	80.0 ± 1.05	80.3 ± 1.50	47.0 ± 1.00	24.1 ± 1.04	36.0 ± 0.58	72.7 ± 1.92	81.0 ± 1.55	50.0 ± 1.82	75.7 ± 1.90
**Colhamine**	2	54.4 ± 1.24	30.1 ± 1.47	20.4 ± 0.12	26.5 ± 1.52	36.7 ± 0.06		14.4 ± 0.18		

Note: (–)-no activity was detected, -reliability of differences *p* < 0.05 compared with the comparison group.

## Data Availability

The data presented in the article can be obtained from the author upon reasonable request.

## Sample Availability

Compound samples are available and kept by the author.
